# An epidemic model with short-lived mixing groups

**DOI:** 10.1007/s00285-022-01822-3

**Published:** 2022-10-31

**Authors:** Frank Ball, Peter Neal

**Affiliations:** School of Mathematical Sciences, University of Nottingham, University Park, Nottingham, NG7 2RD UK

**Keywords:** Branching process, Central limit theorem, Density dependent population process, Final size of epidemic, SIR epidemic, 92D30, 60F05, 60J27, 60J85

## Abstract

Almost all epidemic models make the assumption that infection is driven by the interaction between pairs of individuals, one of whom is infectious and the other of whom is susceptible. However, in society individuals mix in groups of varying sizes, at varying times, allowing one or more infectives to be in close contact with one or more susceptible individuals at a given point in time. In this paper we study the effect of mixing groups beyond pairs on the transmission of an infectious disease in an SIR (susceptible $\rightarrow$ infective $\rightarrow$ recovered) model, both through a branching process approximation for the initial stages of an epidemic with few initial infectives and a functional central limit theorem for the trajectories of the numbers of infectives and susceptibles over time for epidemics with many initial infectives. We also derive central limit theorems for the final size of (i) an epidemic with many initial infectives and (ii) a major outbreak triggered by few initial infectives. We show that, for a given basic reproduction number $R_0$, the distribution of the size of mixing groups has a significant impact on the probability and final size of a major epidemic outbreak. Moreover, the standard pair-based homogeneously mixing epidemic model is shown to represent the worst case scenario, with both the highest probability and the largest final size of a major epidemic.

**Supplementary Information:**

The online version contains supplementary material available at 10.1007/s00285-022-01822-3.

## Introduction

An assumption of the general SIR (susceptible $\rightarrow$ infective $\rightarrow$ recovered) epidemic model is that infection occurs through the interactions of *pairs* of individuals, one of whom is infectious and the other of whom is susceptible. The rate at which infections occur is governed by the rate at which pairs of individuals interact and the probability of transmission during an interaction. These two quantities are often subsumed into a single rate of infectious contacts made by an infective, with any infectious contact with a susceptible resulting in infection. This assumption of the epidemic being driven by pairs of interactions is, at least implicit, in most infectious disease models. The introduction of population heterogeneities in the form of variability in infectivity and susceptibility (eg. Neal 2007), metapopulation (eg. Ball and Clancy 1993), household (eg. Ball et al. 1997) or network (eg. Andersson 1998) models do not depart from the key assumption that infection is between pairs of individuals. One exception is the highly infectious household model, see eg. Becker (1995), where once an individual is infected in a household, their whole household becomes infected. However, this model is often framed in terms of allowing the within-household infection rate $\lambda _L \rightarrow \infty$, and as such represents the limit of the standard household model. Another exception is the Greenwood chain-binomial model, see eg. Bailey (1975), Chapter 14, which is a discrete-time model in which the probability that a given susceptible is infected at a given time depends only on their being infection in the population and not on the number of infectives. However, that assumption is appropriate only for small populations such as households.

The role of *superspreaders* or superspreading events is often mentioned in connection with the emergence of a disease, see eg.  Lau et al. (2017) for Ebola 2014-2015 and  Lewis (2021) for Covid-19. Superspreaders are typically taken to be individuals who are particularly infectious rather than having atypical contacts, see eg. Lloyd-Smith et al. (2005), although in a network setting the infectiousness of an individual is linked to the number of neighbours they have in the network. In many countries one of the most significant NPI (non-pharmaceutical interventions) to reduce the spread of Covid-19 was the introduction of limits on the size of gatherings outside the home, see Flaxman et al. (2020). For example, in the UK, mass gatherings such as sporting events were prohibited and groups were limited in size to 6 individuals, with similar measures employed elsewhere. The motivation behind such an NPI is that gatherings of individuals allow the transmission of a disease from a single infective or small group of infectives to a large group of susceptible individuals. Mass gatherings, such as a football match, typically take place over a short time period, say two hours, in comparison to the infectious period of an individual which will usually be several days. Moreover, individuals infected at a gathering taking place over a time period of two hours are unlikely to become infectious and start transmitting the disease during the same gathering. Therefore, we extend the standard SIR epidemic model to assume that individuals, rather than making contact with single individuals at the points of a Poisson process, are involved in *mixing events* at the points of a Poisson process with mixing events involving $c \ge 2$ individuals. If there is at least one infective and at least one susceptible in a mixing event then there is the possibility of an infection taking place but multiple infections can take place within a single mixing event. It is necessary to model the rate at which mixing events occur and the distribution of the size of mixing events separately from the transmission of the disease within mixing events.

In this paper we consider both the early stages of the epidemic through a branching process approximation and the trajectory of infectives and susceptibles through time in the event of a major epidemic outbreak. By studying the epidemic process as $n \rightarrow \infty$, we are able to compute key epidemic quantities such as the basic reproduction number, $R_0$, and the probability of a major outbreak from the branching process approximation, and the limiting normal distribution for the final size of a major outbreak through a time transformed process. We show that for a given $R_0$, both the probability and mean final size of a major outbreak depend on the distribution of the sizes of mixing events, with the standard SIR epidemic model with pairwise interactions having both the highest probability and the largest mean size of a major outbreak amongst all mixing event distributions. Moreover, for a given $R_0 >1$ and a single initial infective, the probability of a major outbreak can vary between 0 and $1-R_0^{-1}$ (its value for the standard SIR epidemic model) and the mean final size (fraction of the population infected) can vary between $1-R_0^{-1}$ and $\tau _*$, where $\tau _*$ solves $1 - \tau _*= \exp (-R_0 \tau _*)$ and is the mean final size of the standard SIR epidemic model. For example, if $R_0 =2$, the probability of a major outbreak can range between 0 and 0.5 and the mean final size of a major outbreak can range between 0.5 and 0.7968.

The remainder of the paper is structured as follows. In Sect. 2, we define the SIR epidemic model with mixing events. The main results are summarised in Sect. 3, which include the probability of a major outbreak along with the mean and variance of the size of a major outbreak for a general mixing event distribution. These results are asymptotic as the population size $n \rightarrow \infty$. In Sect. 4, we obtain important inequalities which allow us to provide more generally lower and upper bounds for the probability of extinction and the mean final size, along with orderings of mixing event distributions enabling us to show the above-mentioned comparisons with the standard SIR epidemic model. In Sect. 5, we consider logarithmic and geometric mixing event distributions which are amenable to analysis and allow us to obtain explicit expressions for the extinction probability, which further highlight the role of the mixing event distribution. In Sect. 6, we illustrate numerically a selection of the results and use simulations to show their relevance for finite population sizes, *n*. The proofs of the results presented in Sect. 3 are given in Sect. 7. In Sect. 8, we give some concluding comments and discuss possible directions for future work.

## Model

We consider an SIR epidemic in a population of *n* individuals in which infection occurs via mixing events that occur at the points of a Poisson process having rate $n\lambda$. For $i=1,2,\dots$, the $i^\textrm{th}$ mixing events involves $C^{(n)}_i$ individuals, where $C^{(n)}_1, C^{(n)}_2,\dots$ are i.i.d. (independent and identically distributed) realisations of a random variable $C^{(n)}$ which takes values in a subset of $\{2,3,\dots ,n\}$. Suppose $C^{(n)}_i=c$. Then *c* individuals are chosen uniformly at random from the population and at the mixing event any infective present has probability $\pi _c$ of making an infectious contact with any given susceptible present, with all such contacts occurring independently. Mixing events are assumed to be instantaneous. Any susceptible who is contacted by at least one infective at a mixing event becomes infected and remains so for a time that follows an $\textrm{Exp}(\gamma )$ distribution, i.e. an exponential distribution with rate $\gamma$ and hence mean $\gamma ^{-1}$. There is no latent period but newly infected individuals cannot infect susceptibles during the mixing event at which they were infected. All processes and random variables involved in the above model are mutually independent. The process starts with $m_n$ individuals being infective at time $t=0$, with the remaining $n-m_n$ being susceptible, and ends when there is no infective remaining in the population. Denote this epidemic model by ${\mathscr {E}}^{(n)}$.

**Fig. 1:**
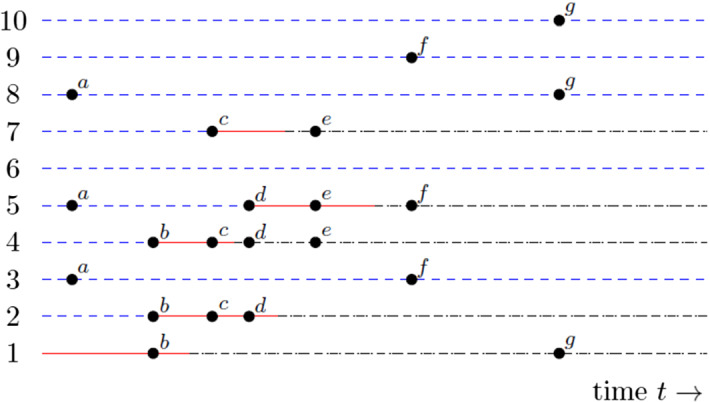
Example of an epidemic outbreak with in a population of size $n=10$, with individuals labelled $1,2,\ldots ,10$, $\textrm{P}(C^{(n)} =3)=1$ and $\pi _3 =1$. Initial condition individual 1 infectious in an otherwise susceptible population. Mixing events labelled $a-g$, circles indicate individuals involved. Susceptible (blue dashed), infectious (red), removed (black dot-dash) (color figure online)

An example of a realisation of an epidemic outbreak is presented in Fig. 1 with $n=10$, $m_n=1$, $\textrm{P}(C^{(n)} =3) =1$ and $\pi _3 =1$, so all mixing events are of size 3 and all susceptibles at a mixing event that contains at least one infective are necessarily infected at that event. There are 7 mixing events, labelled (a) to (g) in chronological order, during the time period shown. Mixing event (a) contains only susceptibles so no new infectives occur. Mixing event (b) involves the initial infective, individual 1, and two susceptibles, individuals 2 and 4, so the latter two become infected. Mixing event (c) involves two infectives, individuals 2 and 4, and one susceptible, individual 7, so individual 7 becomes infected. Mixing event (d) involves one infective, individual 2, one recovered, individual 4, and one susceptible, individual 5, so the latter becomes infected. Mixing event (e) involves one infective, individual 5, and two recovered, individuals 4 and 7, so no new infectives occur. The only remaining infective, individual 5, recovers between mixing events (e) and (f), so the epidemic terminates.

Note that if $\textrm{P}(C^{(n)}=2)=1$, so all mixing groups necessarily consist of 2 individuals, then ${\mathscr {E}}^{(n)}$ is the standard homogeneously mixing stochastic SIR epidemic model, with recovery rate $\gamma$ and individual-to-individual infection rate $\frac{2\lambda \pi _2}{n-1}$. (If there are *s* susceptibles and *i* infectives in the population, the probability that a mixing group of size 2 contains one infective and one susceptible is $$si/\left( {\begin{array}{c}n\\ 2\end{array}}\right) =\frac{2si}{n(n-1)}$$, so the rate at which new infections occur is $n\lambda \times \frac{2si}{n(n-1)} \times \pi _2=\frac{2\lambda \pi _2}{n-1}si$.)

Note also that the groups formed at mixing events are very different from the small mixing groups in for example the households model. In the latter, these groups are permanent, while in the present model their duration is instantaneous.

## Main results

### Introduction

We now present the main results of the paper, which are concerned with the behaviour of the epidemic model introduced in Sect. 2 as the population size $n \rightarrow \infty$. We assume that $C^{(n)}{\mathop {\longrightarrow }\limits ^{\textrm{D}}}C$ as $n \rightarrow \infty$, where ${\mathop {\longrightarrow }\limits ^{\textrm{D}}}$ denotes convergence in distribution and *C* has probability mass function $p_C(c)=\textrm{P}(C=c)$ $(c=2,3,\dots )$. In Sect. 3.2 we consider epidemics with many initial infectives, more precisely the asymptotic regime $n^{-1}m_n \rightarrow \epsilon$ as $n \rightarrow \infty$, where $\epsilon >0$. Thus in the limit as $n \rightarrow \infty$ a strictly positive fraction of the population is initially infected. We give a law of large numbers (LLN), which puts a deterministic approximation of the model on a rigorous footing (Theorem 3.1) and an associated functional central limit theorem (CLT), which describes fluctuations about the limiting deterministic model for large *n* (Theorem 3.2). We also give a CLT for the final size of an epidemic (Theorem 3.3).

In Sect. 3.3 we consider epidemics with few initial infectives, more precisely the asymptotic regime in which $m_n=m$ for all sufficiently large *n*. Thus, for such *n*, each epidemic has *m* initial infectives. Under these conditions, for suitably large *n*, the early stages of the epidemic ${\mathscr {E}}^{(n)}$ can be approximated by a branching process, which we use to determine $R_0$ and the probability that, in the limit $n \rightarrow \infty$, an epidemic becomes established and leads to a major outbreak which infects a strictly positive fraction of the population. We also present a CLT for the size of a major outbreak (Theorem 3.5). The conditions for some of the theorems are quite involved, so in Sect. 3.4 we give simple, easily checkable sufficient conditions which cover most, if not all, practical applications.

### Epidemics with many initial infectives

Throughout this section we assume that $n^{-1}m_n \rightarrow \epsilon$ as $n \rightarrow \infty$, where $\epsilon >0$.

#### Temporal behaviour

For $t \ge 0$, let $S^{(n)}(t)$ and $I^{(n)}(t)$ be respectively the numbers of susceptibles and infectives in ${\mathscr {E}}^{(n)}$ at time *t*. Throughout the paper, for a vector ${\varvec{x}}\in {\mathbb {R}}^d$ for some specified *d*, $\vert {\varvec{x}}\vert$ denotes its Euclidean norm. Further, ${\mathop {\longrightarrow }\limits ^{\textrm{p}}}$ denote convergence in probability.

##### Theorem 3.1

Suppose that $n^{-1}m_n \rightarrow \epsilon$, $C^{(n)}{\mathop {\longrightarrow }\limits ^{\textrm{D}}}C$ and $\textrm{E}[C^{(n)}] \rightarrow \textrm{E}[C]$ as $n \rightarrow \infty$, where $\epsilon >0$ and $\textrm{E}[C^3]<\infty$. Then, for any $t_0>0$,

$$\begin{aligned} \sup _{0 \le t \le t_0} \left| n^{-1}(S^{(n)}(t), I^{(n)}(t))-(x(t), y(t))\right| {\mathop {\longrightarrow }\limits ^{\textrm{p}}}0 \quad \text{ as } n \rightarrow \infty , \end{aligned}$$

where (*x*(*t*), *y*(*t*)) is given by the solution of the following system of ODEs (ordinary differential equations) with initial condition $(x(0), y(0))=(1-\epsilon , \epsilon )$:

3.1 $$\begin{aligned} \dfrac{dx}{dt}=-\lambda x g(y), \qquad \dfrac{dy}{dt}=\lambda x g(y)-\gamma y, \end{aligned}$$

where

3.2 $$\begin{aligned} g(y)=\sum _{c=2}^{\infty } p_C(c)g_c(y), \end{aligned}$$

with

3.3 $$\begin{aligned} g_c(y)=c\left[ 1-(1-y\pi _c)^{c-1}\right] . \end{aligned}$$

The ODEs (3.1) yield a deterministic approximation to the epidemic ${\mathscr {E}}^{(n)}$. The next result is concerned with fluctuations of the sample paths of ${\mathscr {E}}^{(n)}$ about that deterministic limit. Let

3.4 $$\begin{aligned} {\varvec{F}}(x,y)&=(-\lambda x g(y), \lambda x g(y)-\gamma y), \end{aligned}$$

3.5 $$\begin{aligned} \varvec{\partial }{\varvec{F}}(x,y)&=\begin{bmatrix} -\lambda g(y) &{} -\lambda x g'(y) \\ \lambda g(y)&{} \lambda x g'(y)-\gamma \end{bmatrix} \end{aligned}$$

and

3.6 $$\begin{aligned} {\varvec{G}}(x,y)=\begin{bmatrix} \lambda h(x,y) &{} -\lambda h(x,y) \\ -\lambda h(x,y)&{} \lambda h(x,y)+\gamma y \end{bmatrix}, \end{aligned}$$

where

3.7 $$\begin{aligned} h(x,y)=\sum _{c=2}^{\infty } p_C(c)h_c(x,y), \end{aligned}$$

with

3.8 $$\begin{aligned} h_c(x,y){} & {} =c x \left[ 1-(1-y\pi _c)^{c-1}\right] +c(c-1)x^2\left\{ 1-2(1-y\pi _c)^{c-2}\right. \nonumber \\ {}{} & {} \quad \left. +[1-y\pi _c(2-\pi _c)]^{c-2}\right\} . \end{aligned}$$

For $0 \le u \le t < \infty$, let $\varvec{\Phi }(t,u)$ be the solution of the matrix ODE

3.9 $$\begin{aligned} \dfrac{\partial }{\partial t}\varvec{\Phi }(t,u)=\varvec{\partial }{\varvec{F}}(x(t), y(t))\varvec{\Phi }(t,u), \quad \varvec{\Phi }(u,u)={\varvec{I}}, \end{aligned}$$

where ${\varvec{I}}$ denotes the $2 \times 2$ identity matrix. For $n=2,3,\dots$, let $p^{(n)}_C(c)=\textrm{P}(C^{(n)}=c)$ $(c=2,3,\dots )$, so $p^{(n)}_C(c)$ is necessarily zero for $c>n$.

##### Theorem 3.2

Suppose that $\sqrt{n}(n^{-1}m_n-\epsilon ) \rightarrow \epsilon _0$, $C^{(n)}{\mathop {\longrightarrow }\limits ^{\textrm{D}}}C$ and $\textrm{E}[(C^{(n)})^2] \rightarrow \textrm{E}[C^2]$ as $n \rightarrow \infty$, where $\epsilon >0$ and $\textrm{E}[C^4]<\infty$. Suppose also that

3.10 $$\begin{aligned} \lim _{n \rightarrow \infty } \sqrt{n}\sum _{c=2}^{\infty }c \left| p^{(n)}_C(c)-p_C(c)\right| =0. \end{aligned}$$

For $$t \ge 0$$, let

$$\begin{aligned} {\varvec{V}}^{(n)}(t)=\sqrt{n}\left[ n^{-1}(S^{(n)}(t), I^{(n)}(t))-(x(t), y(t))\right] , \end{aligned}$$

where (*x*(*t*), *y*(*t*)) is as in Theorem 3.1. Then

3.11 $$\begin{aligned} \{{\varvec{V}}^{(n)}(t): t \ge 0\} \Rightarrow \{{\varvec{V}}(t): t \ge 0\} \quad \text{ as } n \rightarrow \infty , \end{aligned}$$

where $\Rightarrow$ denotes weak convergence in the space of right-continuous functions $f:[0,\infty ) \rightarrow {\mathbb {R}}^2$ having limits from the left (i.e. càdlàg functions), endowed with the Skorohod metric, and $\{{\varvec{V}}(t):t \ge 0\}$ is a zero-mean Gaussian process with ${\varvec{V}}(0)=(-\epsilon _0,\epsilon _0)$ and covariance function given by

3.12 $$\begin{aligned} \textrm{cov}\left( {\varvec{V}}(t_1), {\varvec{V}}(t_2)\right) =\int _0^{\min (t_1,t_2)}\varvec{\Phi }(t_1,u) {\varvec{G}}(x(u), y(u))\varvec{\Phi }(t_2,u) ^\top \,\textrm{d}u \qquad (t_1,t_2 \ge 0),\nonumber \\ \end{aligned}$$

where $^\top$ denotes transpose.

Note that it follows immediately from (3.12) that

3.13 $$\begin{aligned} \varvec{\Sigma }(t)=\textrm{var}\left( {\varvec{V}}(t)\right) =\int _0^t \varvec{\Phi }(t,u) {\varvec{G}}(x(u),y(u))\varvec{\Phi }(t,u) ^\top \,\textrm{d}u. \end{aligned}$$

Differentiating (3.13) and using (3.9) yields that $\varvec{\Sigma }(t)$ satisfies the matrix ODE

3.14 $$\begin{aligned} \dfrac{d\varvec{\Sigma }}{dt}={\varvec{G}}(x(t),y(t))+\varvec{\partial }{\varvec{F}}(x(t), y(t)) \varvec{\Sigma }+\varvec{\Sigma }[\varvec{\partial }{\varvec{F}}(x(t), y(t))]^{\top }, \end{aligned}$$

with initial condition $\varvec{\Sigma }(0)={\varvec{0}}$, the $2 \times 2$ matrix of zeros. Thus $\varvec{\Sigma }(t)$ can be computed by numerically solving the ODEs (3.1) and (3.14) simultaneously.

#### Final outcome

Let $T^{(n)}=n-S^{(n)}(\infty )$ be the total number of individuals infected during the epidemic (including the initial infectives), i.e. its final size. As detailed in Sect. 7.4, we prove a CLT for $T^{(n)}$ by considering a random time-scale transformation of $\{(S^{(n)}(t),I^{(n)}(t)): t \ge 0\}$ in which the clock is slowed down by a factor $n^{-1}I^{(n)}(t)$. This leads to the time-transformed deterministic model given by

3.15 $$\begin{aligned} \dfrac{d{\tilde{x}}}{dt}=-\lambda {\tilde{x}}{\tilde{g}}({\tilde{y}}),\quad \dfrac{d{\tilde{y}}}{dt}=\lambda {\tilde{x}}{\tilde{g}}({\tilde{y}})-\gamma , \quad ({\tilde{x}}(0),{\tilde{y}}(0))=(1-\epsilon ,\epsilon ), \end{aligned}$$

where

3.16 $$\begin{aligned} {\tilde{g}}(y)= {\left\{ \begin{array}{ll} y^{-1}g(y) &{} \text { if } 0<y \le 1,\\ \sum _{c=2}^{\infty }p_C(c) c(c-1) \pi _c &{} \text{ if } y=0. \end{array}\right. } \end{aligned}$$

Adding the differential equations (3.15) and solving yields

3.17 $$\begin{aligned} {\tilde{x}}(t)=1-{\tilde{y}}(t) -\gamma t \qquad (t \ge 0), \end{aligned}$$

so $${\tilde{y}}(t)$$ satisfies

3.18 $$\begin{aligned} \dfrac{d{\tilde{y}}}{dt}=\lambda (1-{\tilde{y}}-\gamma t) {\tilde{g}}({\tilde{y}})-\gamma ,\qquad {\tilde{y}}(0)=\epsilon . \end{aligned}$$

The time transformation does not change the final outcome of the epidemic. Let ${\tilde{\tau }}_{\epsilon }=\inf \{t>0:{\tilde{y}}(t)=0\}$, so using (3.17), ${\tilde{x}}({\tilde{\tau }}_{\epsilon })=1-\gamma {\tilde{\tau }}_{\epsilon }$. Note that $x(\infty )={\tilde{x}}({\tilde{\tau }}_{\epsilon })$, so the fraction of the population infected (including the initial infectives) in the deterministic epidemic given by (3.1), with $(x(0), y(0))=(1-\epsilon , \epsilon )$, is $\gamma {\tilde{\tau }}_{\epsilon }$. The CLT for $T^{(n)}$ is proved by deriving an analogous functional CLT to Theorem 3.2 for the time-changed process and then considering an associated crossing problem. Before stating the theorem some more notation is required.

Let

3.19 $$\begin{aligned} d_{\epsilon }=\frac{R_0(1-\gamma {\tilde{\tau }}_{\epsilon })}{1-R_0(1-\gamma {\tilde{\tau }}_{\epsilon })}, \end{aligned}$$

where $R_0$, the basic reproduction number (see Sect. 3.3), is given by

3.20 $$\begin{aligned} R_0=\frac{\lambda }{\gamma } \sum _{c=2}^{\infty } p_C(c)c(c-1)\pi _c. \end{aligned}$$

Let $({\tilde{\sigma }}_{S,\epsilon }^2(t),{\tilde{\sigma }}_{SI,\epsilon }(t), {\tilde{\sigma }}_{I,\epsilon }^2(t))$ be the solution of the following system of ODEs with initial condition $({\tilde{\sigma }}_{S}^2(0),{\tilde{\sigma }}_{SI}(0), {\tilde{\sigma }}_{I}^2(0))=(0,0,0)$:

3.21 $$\begin{aligned} \dfrac{d{\tilde{\sigma }}_S^2}{dt}&=\lambda {\tilde{h}}({\tilde{x}}(t),{\tilde{y}}(t))-2\lambda [{\tilde{g}}({\tilde{y}}(t)){\tilde{\sigma }}_S^2+{\tilde{x}}(t){\tilde{g}}'({\tilde{y}}(t)){\tilde{\sigma }}_{SI}], \end{aligned}$$

3.22 $$\begin{aligned} \dfrac{d{\tilde{\sigma }}_{SI}}{dt}&=-\lambda {\tilde{h}}({\tilde{x}}(t),{\tilde{y}}(t))\nonumber \\&\quad +\lambda \left\{ {\tilde{g}}({\tilde{y}}(t)){\tilde{\sigma }}_S^2+[{\tilde{x}}(t){\tilde{g}}'({\tilde{y}}(t))-{\tilde{g}}({\tilde{y}}(t))]{\tilde{\sigma }}_{SI} -{\tilde{x}}(t){\tilde{g}}'({\tilde{y}}(t)){\tilde{\sigma }}_I^2\right\} , \end{aligned}$$

3.23 $$\begin{aligned} \dfrac{d{\tilde{\sigma }}_I^2}{dt}&=\gamma +\lambda {\tilde{h}}({\tilde{x}}(t),{\tilde{y}}(t))+2\lambda [{\tilde{g}}({\tilde{y}}(t)){\tilde{\sigma }}_{SI}+{\tilde{x}}(t){\tilde{g}}'({\tilde{y}}(t)){\tilde{\sigma }}_I^2], \end{aligned}$$

where $({\tilde{x}}(t),{\tilde{y}}(t))$ are given by (3.17) and (3.18), and

3.24 $$\begin{aligned} {\tilde{h}}(x,y)= {\left\{ \begin{array}{ll} y^{-1}h(x,y) &{} \text { if } 0<y \le 1,\\ \sum _{c=2}^{\infty }p_C(c) c(c-1) \pi _c x[1+(c-2)\pi _c x] &{} \text{ if } y=0. \end{array}\right. } \end{aligned}$$

Let

$$\begin{aligned} \sigma _T^2(\epsilon )= {\tilde{\sigma }}_{S,\epsilon }^2({\tilde{\tau }}_{\epsilon })-2d_{\epsilon } {\tilde{\sigma }}_{SI,\epsilon }({\tilde{\tau }}_{\epsilon })+d_{\epsilon }^2 {\tilde{\sigma }}_{I,\epsilon }^2({\tilde{\tau }}_{\epsilon }). \end{aligned}$$

For future reference, define ${\tilde{\tau }}_0$ and $\sigma _T^2(0)$ by setting $\epsilon =0$ in the above.

For $i,j=0,1,\dots$ and $y_0>0$, let

3.25 $$\begin{aligned} {\tilde{S}}_1(i,j,y_0)=\sum _{c=2}^{\infty } c^i \pi _c^j (1+\pi _cy_0)^c p_C(c) \end{aligned}$$

and

3.26 $$\begin{aligned} {\tilde{S}}_2(i,j,y_0)=\sum _{c=2}^{\infty } c^i \pi _c^j [1+\pi _c(2-\pi _c)y_0]^c p_C(c). \end{aligned}$$

The CLT assumes that the following conditions hold:

3.27 $$\begin{aligned}{} & {} \sum _{c=2}^{\infty } \pi _c c^5 p_C(c)< \infty , \end{aligned}$$

3.28 $$\begin{aligned}{} & {} \lim _{n \rightarrow \infty } \sum _{c=2}^{\infty } \pi _c c^3 p^{(n)}_C(c)=\sum _{c=2}^{\infty } \pi _c c^3 p_C(c), \end{aligned}$$

3.29 $$\begin{aligned}{} & {} \lim _{n \rightarrow \infty } \sqrt{n}\sum _{c=2}^{\infty }\pi _c c^2 \left| p^{(n)}_C(c)-p_C(c)\right| =0, \end{aligned}$$

and there exists $y_0>0$ is such that

3.30 $$\begin{aligned} {\tilde{S}}_1(i,i-1,y_0)< \infty \quad (i=1,2,3) \quad \text{ and }\quad {\tilde{S}}_2(i,i-2,y_0)< \infty \quad (i=2,3).\nonumber \\ \end{aligned}$$

##### Theorem 3.3

Suppose that $\sqrt{n}(n^{-1}m_n-\epsilon ) \rightarrow \epsilon _0$ and $C^{(n)}{\mathop {\longrightarrow }\limits ^{\textrm{D}}}C$ as $n \rightarrow \infty$, where $\epsilon >0$, and conditions (3.27)–(3.30) are satisfied. Then

$$\begin{aligned} \sqrt{n}\left( n^{-1}T^{(n)}-\gamma {\tilde{\tau }}_{\epsilon }\right) {\mathop {\longrightarrow }\limits ^{\textrm{D}}}\textrm{N}(0,\sigma _T^2(\epsilon )), \end{aligned}$$

where $\textrm{N}(0,\sigma _T^2(\epsilon ))$ denotes a normal distribution with mean 0 and variance $\sigma _T^2(\epsilon )$.

### Epidemics with few initial infectives

Throughout this section, we assume that $m_n=m$ for all sufficiently large *n*. Consider a typical infective, $i_*$ say, in the early phase of the epidemic ${\mathscr {E}}^{(n)}$. The probability that a mixing event of size *c* involves $i_*$ is $\frac{c}{n}$. Mixing events occur at rate $n\lambda$ and have sizes that are i.i.d. realisations of $C^{(n)}$, so mixing events that involve $i_*$ occur at rate $\lambda \mu ^{(n)}_C$, where $\mu ^{(n)}_C=\textrm{E}[C^{(n)}]$, and, for $c=2,3,\dots ,n$, the probability that a mixing event is of size *c* given that it includes $i_*$ is $c p^{(n)}_C(c)/\mu ^{(n)}_C$. Moreover, since mixing events are formed by choosing individuals uniformly at random from the population, with probability close to 1, every mixing event involving $i_*$ consists otherwise entirely of susceptibles. By considering the limits of the above quantities as $n \rightarrow \infty$, the early phase of the epidemic ${\mathscr {E}}^{(n)}$ can be approximated by the following branching process, denoted by ${\mathscr {B}}$, in which individuals and birth events correspond respectively to infectives and mixing events in the epidemic process ${\mathscr {E}}^{(n)}$.

Individuals in the branching process ${\mathscr {B}}$ behave independently. A given individual, $i_*$ say, has lifetime $L \sim \textrm{Exp}(\gamma )$, during which they have birth events at the points of a Poisson process having rate $\lambda \mu _C$. The numbers of offspring individual $i_*$ produces at their successive birth events, ${\tilde{Z}}_1, {\tilde{Z}}_2, \dots$, are i.i.d. copies of a random variable ${\tilde{Z}}$ having distribution defined as follows. The size of a typical mixing event involving $i_*$ is distributed as ${\tilde{C}}$, the size-biased version of *C*, having probability mass function

3.31 $$\begin{aligned} p_{{\tilde{C}}}(c)=\textrm{P}({\tilde{C}}=c)=\mu _C^{-1}cp_C(c) \qquad (c=2,3,\dots ). \end{aligned}$$

If the mixing event is of size *c* then there are $c-1$ susceptibles present, each of whom is infected independently with probability $\pi _c$ by $i_*$. Thus, ${\tilde{Z}}$ is distributed as a mixture of $\textrm{Bin}(c-1, \pi _c)$ $(c=2,3,\dots )$ random variables with respective mixing probabilities $p_{{\tilde{C}}}(c)$ $(c=2,3,\dots )$, Note that an individual may have no offspring at a birth event.

Let *R* denote the total number of offspring a typical individual has in ${\mathscr {B}}$. Then

3.32 $$\begin{aligned} R={\tilde{Z}}_1+{\tilde{Z}}_2+\dots +{\tilde{Z}}_G, \end{aligned}$$

where *G* is the number of birth events that an individual has in their lifetime and $R=0$ if $G=0$. Further, *G* is independent of ${\tilde{Z}}_1, {\tilde{Z}}_2, \dots$ and has the geometric distribution

3.33 $$\begin{aligned} \textrm{P}(G=k)=\frac{\gamma }{\gamma +\lambda \mu _C}\left( \frac{\lambda \mu _C}{\gamma +\lambda \mu _C}\right) ^k \qquad (k=0,1,\dots ). \end{aligned}$$

Hence,

3.34 $$\begin{aligned} \textrm{E}[R]=\textrm{E}[G]\textrm{E}[{\tilde{Z}}]=\frac{\lambda \mu _C}{\gamma } \textrm{E}[({\tilde{C}}-1)\pi _{{\tilde{C}}}]=\frac{\lambda }{\gamma }\sum _{c=2}^{\infty }\pi _c c(c-1)p_C(c), \end{aligned}$$

using (3.31). Note that $E[R]=R_0$, where $R_0$ is given by (3.20). Hence, as stated in Sect. 3.2.2, $R_0$ is the basic reproduction number of the epidemic ${\mathscr {E}}^{(n)}$.

Let *z* denote the extinction probability of ${\mathscr {B}}$ given that initially there is one individual. Then from standard branching process theory, *z* is given by the smallest solution in [0, 1] of $f_R(s)=s$, where $f_R$ is the probability-generating function of *R*; moreover, $z<1$ if and only if $R_0>1$. (Throughout the paper, if *X* is a random variable taking values in ${\mathbb {Z}}_+$ then $f_X$ denotes its probability-generating function, viz. $f_X(s)=\textrm{E}[s^X]$ $(0 \le s \le 1)$.) It follows from (3.32) and (3.33) that

3.35 $$\begin{aligned} f_R(s)=\sum _{k=0}^{\infty }\frac{\gamma }{\gamma +\lambda \mu _C}\left( \frac{\lambda \mu _C}{\gamma +\lambda \mu _C}\right) ^k\left( f_{{\tilde{Z}}}(s)\right) ^k=\frac{\gamma }{\gamma +\lambda \mu _C\left( 1-f_{{\tilde{Z}}}(s)\right) },\nonumber \\ \end{aligned}$$

where

3.36 $$\begin{aligned} f_{{\tilde{Z}}}(s)=\sum _{c=2}^{\infty }p_{{\tilde{C}}}(c)(1-\pi _c+\pi _c s)^{c-1} =\frac{1}{\mu _C}\sum _{c=2}^{\infty }p_C(c)c (1-\pi _c+\pi _c s)^{c-1}.\nonumber \\ \end{aligned}$$

The Malthusian parameter (exponential growth rate) *r* of the branching process ${\mathscr {B}}$ is easily obtained since the mean rate an individual produces offspring *t* time units after their birth is $\textrm{P}(L>t) \lambda \mu _C \textrm{E}[{\tilde{Z}}_1]=\gamma \textrm{e}^{-\gamma t} R_0$ $(t > 0)$, so the Lotka-Euler equation is $\int _0^{\infty }\textrm{e}^{-rt} \gamma \textrm{e}^{-\gamma t} R_0 \,\textrm{d}t=1$, yielding

$$\begin{aligned} r=\gamma (R_0-1). \end{aligned}$$

Note that if $R_0$ and $\gamma$ are held fixed, then *r* is the same for all corresponding choices of the distribution of *C* and $\pi _k$ $(k=2,3,\dots )$. In particular, under these conditions, the early exponential growth of an epidemic that takes off is the same as that of a standard homogeneously mixing epidemic.

The formulae for $R_0$ and $f_R(s)$ simplify when the infection probability $\pi _c$ is independent of *c*, say $\pi _c=\pi$ for all *c*. In particular, (3.34) then yields

3.37 $$\begin{aligned} R_0=\frac{\lambda \pi }{\gamma } \textrm{E}[C(C-1)]. \end{aligned}$$

The approximating branching process ${\mathscr {B}}$ can be put on a rigorous footing by theorems concerning convergence of ${\mathscr {E}}^{(n)}$ to ${\mathscr {B}}$ as $n \rightarrow \infty$. However, their proofs are long and are presented separately in Ball and Neal (under review). The usual approach to proving such theorems (see e.g. Ball and Donnelly 1995) is to construct sample paths of ${\mathscr {E}}^{(n)}$ for each *n* from those of the limiting branching process ${\mathscr {B}}$. That approach is not easily implemented in the present setting as mixing groups induce dependencies between infectives. The following theorem, that is used in the proof of Theorem 3.5 below, is proved in Ball and Neal (under review).

#### Theorem 3.4

- Suppose that $m_n=m$ for all sufficiently large *n*, $C^{(n)}{\mathop {\longrightarrow }\limits ^{\textrm{D}}}C$ and $\textrm{E}[(C^{(n)})^2] \rightarrow \textrm{E}[C^2]$ as $n \rightarrow \infty$, where $E[C^2]<\infty$, and conditions (3.10) and (3.28) hold. Then
  3.38 $$\begin{aligned} \textrm{P}(T^{(n)}\ge \log n) \rightarrow 1-z^m \quad \text{ as } n \rightarrow \infty . \end{aligned}$$
- If also $R_0>1$ then there exists $\delta >0$ such that
  3.39 $$\begin{aligned} \textrm{P}(T^{(n)}\ge \delta n \mid T^{(n)}\ge \log n) \rightarrow 1 \quad \text{ as } n \rightarrow \infty . \end{aligned}$$

In view of Theorem 3.4(a), we define a *major outbreak* to be one whose final size is at least $\log n$. Theorem 3.4(b) implies that a major outbreak infects at least a fraction $\delta$ of the population with probability tending to one as $n \rightarrow \infty$. However, $\delta$ depends on the parameters of the epidemic ${\mathscr {E}}^{(n)}$ and can be arbitrarily close to 0. The final result in this section gives a CLT for the size of a major outbreak. Recall the notation ${\tilde{\tau }}_0$ and $\sigma _T^2(0)$ introduced just after (3.24).

#### Theorem 3.5

Suppose that $R_0>1$, $m_n=m$ for all sufficiently large *n*, $C^{(n)}{\mathop {\longrightarrow }\limits ^{\textrm{D}}}C$ as $n \rightarrow \infty$, and conditions (3.27)–(3.30) are satisfied. Then

$$\begin{aligned} \sqrt{n}\left( n^{-1}T^{(n)}-\gamma {\tilde{\tau }}_0\right) \left| \right. T^{(n)}\ge \log n {\mathop {\longrightarrow }\limits ^{\textrm{D}}}\textrm{N}(0,\sigma _T^2(0)). \end{aligned}$$

### Sufficient conditions for theorems

Simpler, easily checkable sufficient conditions for the above theorems are proved in Appendix A in the Supplementary Information. If there is a maximum mixing group size, $c_*$ say, so $\textrm{P}(C^{(n)}>c_*)=0$ for all *n*, then the conditions concerning $C^{(n)}$ and *C* of all of the theorems are satisfied if $\lim _{n \rightarrow \infty } \sqrt{n}[p^{(n)}_C(c)-p_C(c)]=0$ $(c=2,3,\dots , c_*)$. Two natural choices for the distribution of $C^{(n)}$ when there is no maximum mixing group size are $C^{(n)}{\mathop {=}\limits ^{D}}\min (C, n)$ and $C^{(n)}{\mathop {=}\limits ^{D}}(C\vert C \le n)$, where ${\mathop {=}\limits ^{D}}$ denotes equal in distribution. In both of these cases the conditions concerning $C^{(n)}$ and *C* are satisfied if $f_C(s_1)< \infty$ for some $s_1>1$. This condition requires that *C* has finite moments of all orders but it holds for all choices of infection probabilities $\pi _c$ $(c=2,3,\dots )$. If these probabilities satisfy $\pi _c\le \frac{\zeta }{c}$ for all sufficiently large *c*, for some $\zeta \in (0, \infty )$, then $\textrm{E}[C^4]<\infty$ is sufficient for the conditions concerning $C^{(n)}$ and *C*.

## Model comparisons

### Constant mixing event infection probability $\pi _c$

In this subsection we assume that $\pi _c=\pi$ for all $c=2,3,\dots$. Recall that *z* denotes the extinction probability of the branching process ${\mathscr {B}}$. Further, for $\epsilon \in (0,1]$, for a sequence of epidemics $({\mathscr {E}}^{(n)})$ satisfying $\lim _{n \rightarrow \infty } n^{-1}m_n = \epsilon$, ${\tilde{\tau }}_{\epsilon }$ is the fraction of the population that is infected by the epidemic ${\mathscr {E}}^{(n)}$ in the limit as $n \rightarrow \infty$. If instead, $m_n=m$ for all sufficiently large *n*, then ${\tilde{\tau }}_{0}$ is the fraction of the population that is infected by a major outbreak in the limit as $n \rightarrow \infty$.

The following proposition facilitates comparison of the above properties between epidemics when $R_0$ is held fixed and also calculation/computation of some of these properties in the special cases considered in Sect. 5. Suppose that $\textrm{E}[C^2] < \infty$ and let ${\hat{C}}$ be a random variable having probability mass function

4.1 $$\begin{aligned} p_{{\hat{C}}}(c)=\textrm{P}({\hat{C}}=c)=\frac{c(c-1)p_C(c)}{\textrm{E}[C(C-1)]} \qquad (c=2,3,\dots ). \end{aligned}$$

#### Proposition 4.1

For $$0 \le y \le 1$$, let

4.2 $$\begin{aligned} U(y)=\frac{R_0}{\pi } \int _{1-\pi y}^1 f_{{\hat{C}}-2}(x)\,\textrm{d}x =R_0 \int _0^y f_{{\hat{C}}-2}(1-\pi u)\,\textrm{d}u. \end{aligned}$$

- The extinction probability $z=1-w$, where *w* is the greatest solution in [0, 1] of $(1-y)U(y)=y$.
- For $0 \le \epsilon <1$, ${\tilde{\tau }}_{\epsilon }=\inf \{t>0:{\bar{y}}(t)=0\}$, where ${\bar{y}}(t)$ $(t \ge 0)$ is the solution of
  4.3 $$\begin{aligned} \dfrac{d{\bar{y}}}{dt}=(1-{\bar{y}}-t) \frac{1}{{\bar{y}}}U({\bar{y}})-1,\qquad {\bar{y}}(0)=\epsilon . \end{aligned}$$

#### Proof

(a) It follows from (3.35) that $f_R(s)=(1+V(s))^{-1}$, where

$$\begin{aligned} V(s)=\frac{\lambda \mu _C}{\gamma }(1-f_{{\tilde{Z}}}(s))=\frac{R_0}{\pi } \sum _{c=2}^{\infty }\frac{cp_C(c)}{\textrm{E}[C(C-1)]}\left[ 1-(1-\pi +\pi s)^{c-1}\right] , \end{aligned}$$

using (3.36) and (3.37). Now $1-(1-\pi +\pi s)^{c-1}=(c-1)\int _{1-\pi +\pi s}^{1}x^{c-2}\,\textrm{d}x$, so

$$\begin{aligned} V(s)=\frac{R_0}{\pi } \sum _{c=2}^{\infty } p_{{\hat{C}}}(c)\int _{1-\pi +\pi s}^{1}x^{c-2}\,\textrm{d}x=\frac{R_0}{\pi } \int _{1-\pi +\pi s}^{1}f_{{\hat{C}}-2}(x)\,\textrm{d}x=U(1-s). \end{aligned}$$

Part (a) follows since *z* is the smallest solution in [0, 1] of $f_R(s)=s$. (The second expression for *U*(*y*) in (4.2) follows from the first via the substitution $u=(1-x)/\pi$.)

(b) Assume without loss of generality that the time scale is chosen so that $\gamma =1$, whence $R_0=\lambda \pi \textrm{E}[C(C-1)]$. Thus, ${\tilde{\tau }}_{\epsilon }=\inf \{t>0:{\tilde{y}}(t)=0\}$, where ${\tilde{y}}(t)$ $(t \ge 0)$ satisfies (3.18) with $\gamma =1$, and part (b) follows if $\lambda {\tilde{g}}({\tilde{y}})=\frac{1}{{\tilde{y}}}U({\tilde{y}})$ $({\tilde{y}}\ge 0)$. Using (3.2), (3.3) and (3.16),

$$\begin{aligned} \lambda {\tilde{g}}({\tilde{y}})=\frac{\lambda }{{\tilde{y}}}\sum _{c=2}^{\infty }cp_C(c)\left[ 1-(1-\pi {\tilde{y}})^{c-1}\right] =\frac{R_0}{\pi {\tilde{y}}} \sum _{c=2}^{\infty }\frac{cp_C(c)}{\textrm{E}[C(C-1)]}\left[ 1-(1-\pi {\tilde{y}})^{c-1}\right] . \end{aligned}$$

The same argument as in the proof of part (a) now shows that $\lambda {\tilde{g}}({\tilde{y}})=\frac{1}{{\tilde{y}}}U({\tilde{y}})$, as required. $\square$

Let ${\mathscr {D}}$ denote the space of random variables taking values in $\{2,3,\dots \}$ and, for $c=3,4,\dots$, let ${\mathscr {D}}_c$ be the subspace of ${\mathscr {D}}$ consisting of random variables that take values in $\{2,3,\dots ,c\}$. Recall PGF ordering (${\mathop {\le }\limits ^{g}}$) of random variables. For random variables $C, C' \in {\mathscr {D}}$, $C {\mathop {\le }\limits ^{g}}C'$ if $f_C(s) \ge f_{C'}(s)$ for all $s \in [0,1]$. For $c=2,3,\dots$, let $\textrm{Const}(c)$ denote the distribution consisting of a unit mass at *c*.

Suppose that $C \sim \textrm{Const}(2)$. Then $U(y)=R_0y$ $(0 \le y \le 1)$ and it follows using Proposition 4.1 that $z=\min (\frac{1}{R_0},1)$. Further, (4.3) becomes

4.4 $$\begin{aligned} \dfrac{d{\bar{y}}}{dt}=R_0(1-{\bar{y}}-t)-1,\qquad {\bar{y}}(0)=\epsilon , \end{aligned}$$

having solution

4.5 $$\begin{aligned} {\bar{y}}(t)=1-t-(1-\epsilon )\textrm{e}^{-R_0t}\quad (t \ge 0). \end{aligned}$$

Thus ${\tilde{\tau }}_{\epsilon }=\inf \{t>0:1-t=(1-\epsilon )\textrm{e}^{-R_0 t}\}$ ($={\tilde{\tau }}_{\epsilon }^*(R_0)$, say). Of course these results coincide with those of the standard homogeneously mixing SIR epidemic model.

Note that Proposition 4.1 implies that *z* and ${\tilde{\tau }}_{\epsilon }$ $(\epsilon \in [0,1)$) are determined by $(R_0, C, \pi )$, so write $z=z(R_0, C, \pi )$ and ${\tilde{\tau }}_{\epsilon }={\tilde{\tau }}_{\epsilon }(R_0, C, \pi )$, where it is assumed implicitly that $R_0>1$ if $\epsilon =0$.

#### Theorem 4.1

- For any $$C \in {\mathscr {D}}$$,
  (i) if $\pi > \pi '$ then $z(R_0, C, \pi ) \ge z(R_0, C, \pi ')$ and ${\tilde{\tau }}_{\epsilon }(R_0, C, \pi )\le {\tilde{\tau }}_{\epsilon }(R_0, C, \pi ')$, with strict inequalities if $\textrm{P}(C>2)>0$;
  (ii) $z(R_0, C, \pi ) \rightarrow \min (\frac{1}{R_0},1)$ and ${\tilde{\tau }}_{\epsilon }(R_0, C, \pi ) \rightarrow {\tilde{\tau }}_{\epsilon }^*(R_0)$ as $\pi \downarrow 0$.
- Suppose that $\pi \in (0,1]$ is fixed.
  (i) Suppose that $C, C' \in {\mathscr {D}}$, $C {\mathop {\ne }\limits ^{D}}C'$ and ${\hat{C}}' {\mathop {\le }\limits ^{g}}{\hat{C}}$. Then $z(R_0, C, \pi ) \ge z(R_0, C', \pi )$, with strict inequality if $R_0>1$, and ${\tilde{\tau }}_{\epsilon }(R_0, C, \pi ) < {\tilde{\tau }}_{\epsilon }(R_0, C', \pi )$.
  (ii) For all $C \in {\mathscr {D}}$ satisfying $\textrm{P}(C=2)<1$, $z(R_0, C, \pi ) \ge \min \left( \frac{1}{R_0},1\right)$, with strict inequality if $R_0>1$, and ${\tilde{\tau }}_{\epsilon }(R_0, C, \pi ) < {\tilde{\tau }}_{\epsilon }^*(R_0)$.
  (iii) Fix $c \ge 3$ and let $C^* \sim \text {Const}(c)$. For all $C \in {\mathscr {D}}_c {\setminus }\{C^*\}$, $z(R_0, C, \pi ) \le z(R_0, C^*, \pi )$, with strict inequality if $R_0>1$, and ${\tilde{\tau }}_{\epsilon }(R_0, C, \pi ) > {\tilde{\tau }}_{\epsilon }(R_0, C^*, \pi )$.

#### Proof

(a) Write *U*(*y*) as $U(y, C, \pi )$ to show explicitly its dependence on $(C,\pi )$. By Proposition 4.1(a), $z(R_0, C, \pi )=1-w(R_0, C, \pi )$, where $w(R_0, C, \pi )$ is the greatest solution in [0, 1] of $(1-y)U(y, C, \pi )=y$. If $R_0 \le 1$, then $z(R_0, C, \pi ) = z(R_0, C, \pi ')=1$. Suppose $R_0>1$ and $\pi >\pi '$. Since $f_{{\hat{C}}-2}$ is increasing on [0, 1], it follows immediately from the second equality in (4.2) that $U(y, C, \pi ) \le U(y, C, \pi ')$ $(0 \le y \le 1)$, with strict inequality $y>0$ provided $\textrm{P}(C>2)>0$. Hence, $w(R_0, C, \pi ) \le w(R_0, C, \pi ')$, with strict inequality if $\textrm{P}(C>2)>0$, and the inequality for *z* follows.

For $0 \le \epsilon <1$, let ${\bar{y}}_{\epsilon }(t, \pi )$ $(t \ge 0)$ be the solution of (4.3) with $U({\bar{y}})$ replaced by $U({\bar{y}}, \epsilon )$. Since $U(y, C, \pi ) \le U(y, C, \pi ')$ $(0 \le y \le 1)$, it follows from (4.3) that ${\bar{y}}_{\epsilon }(t, \pi ) \le {\bar{y}}_{\epsilon }(t, \pi ')$, for $0 \le t \le \tau (R_0, C, \pi )$, so ${\tilde{\tau }}(R_0, C, \pi )\le {\tilde{\tau }}(R_0, C, \pi ')$. Further, ${\tilde{\tau }}(R_0, C, \pi )< {\tilde{\tau }}(R_0, C, \pi ')$ if $\textrm{P}(C>2)>0$, since then $U(y, C, \pi ) < U(y, C, \pi ')$ $(0 < y \le 1)$.

Part (a)(ii) is proved by noting from (4.2) that $U(y, C, \pi ) \rightarrow R_0 y$ $(0 \le y \le 1)$ as $\pi \downarrow 0$.

(b) Suppose that ${\hat{C}}' {\mathop {\le }\limits ^{g}}{\hat{C}}$. Then (4.2) implies that $U(y, C, \pi ) \le U(y, C', \pi )$ $(0 \le y \le 1)$, and the proof of part (b)(i) parallels that of part (a)(i) in the obvious fashion. Parts (b)(ii),(iii) are proved by noting that ${\hat{C}}_* {\mathop {\le }\limits ^{g}}{\hat{C}} {\mathop {\le }\limits ^{g}}{\hat{C}}^*$, for all $C \in {\mathscr {D}}_c$, where $C_* \sim \text {Const}(2)$. $\square$

Theorem 4.1(a) shows that if $R_0>1$ and the event size distribution *C* are held fixed, then the epidemic increases as the infection probability $\pi$ decreases, in the sense that both the probability of and the fraction of the population infected by a major outbreak increase. Moreover, as $\pi \downarrow 0$, these properties tend to the corresponding properties of the standard homogeneously mixing SIR model. (If $C \sim \text {Const}(2)$, so all mixing events are of size 2, and $R_0$ is held fixed, then the distribution of the final outcome of the epidemic is independent of $\pi \in (0,1]$.) Theorem 4.1(b) shows that if $R_0>1$ and $\pi \in (0, 1]$ are held fixed, then the epidemic tends to decrease (in the above sense) as the mixing event size *C* increases. Moreover, the standard homogeneously mixing SIR model necessarily provides an upper bound for the model with mixing events. Furthermore, if there is a maximum mixing event size, $c_{\max }$ say, then the model in which mixing events have size $c_{\max }$ yields a lower bound.

### Varying mixing event infection probability $\pi _c$

Let $\varvec{\pi }=(\pi _2, \pi _3, \dots )$. As before, we assume that $R_0$ is held fixed. Note that *z* and ${\tilde{\tau }}_{\epsilon }$ are each determined by $(R_0, C, \varvec{\pi })$, so we write $z=z(R_0, C, \varvec{\pi })$ and ${\tilde{\tau }}_{\epsilon }=\tau _{\epsilon }(R_0, C, \varvec{\pi })$. Theorem 4.2 below provides bounds on these quantities. For $R_0>0$, define $f_{R_0}:[0, \infty ) \rightarrow [0, \infty )$ by $f_{R_0}(t)=t-\frac{1}{R_0}(1-\textrm{e}^{-R_0 t})$. Note that $f_{R_0}$ is monotonically increasing and let $f_{R_0}^{-1}$ be the inverse function of $f_{R_0}$. As is apparent from the proof of Theorem 4.2, for $R_0>1$ and $\epsilon \in (0, 1-R_0^{-1})$, $f_{R_0}^{-1}(\epsilon )$ is the size (including the initial infectives) of the standard deterministic homogeneously mixing SIR epidemic having basic reproduction number $R_0$, when the initial fractions susceptible and infected are $R_0^{-1}$ and $\epsilon$, respectively. Let ${\mathscr {P}}$ denote the set of all possible $\varvec{\pi }$.

#### Theorem 4.2

- For any $(C, \varvec{\pi }) \in {\mathscr {D}}\times {\mathscr {P}}$ with $\sum _{c=2}^{\infty } p_C(c)\pi _c>0$,
  $$\begin{aligned} z(R_0, C, \varvec{\pi }) \ge \min (R_0^{-1},1) \quad \text {and}\quad {\tilde{\tau }}_{\epsilon }(R_0, C, \varvec{\pi }) \le \tau _{\epsilon }^*(R_0)\quad (\epsilon \in [0,1)). \end{aligned}$$
- Let
  $$\begin{aligned} a(C, \varvec{\pi })= \frac{\mu _C}{\sum _{c=2}^{\infty } p_C(c)c(c-1)\pi _c}. \end{aligned}$$
  Then, for any $(C, \varvec{\pi }) \in {\mathscr {D}}\times {\mathscr {P}}$ with $R_0>1$,
  4.6 $$\begin{aligned} 1-R_0^{-1} < {\tilde{\tau }}_0(R_0, C, \varvec{\pi }) \le 1-R_0^{-1}+f_{R_0}^{-1}(a(C, \varvec{\pi })R_0)-a(C, \varvec{\pi })R_0\nonumber \\ \end{aligned}$$
  and
  4.7 $$\begin{aligned} \frac{1}{1+a(C, \varvec{\pi })R_0}< z <1. \end{aligned}$$

#### Proof

Throughout the proof we assume without loss of generality that $\gamma =1$.

(a) Using (3.36), for $$0 \le s \le 1$$,

$$\begin{aligned} f_{{\tilde{Z}}}(s) \ge 1-\frac{1-s}{\mu _C}\sum _{c=2}^{\infty }p_C(c)c (c-1)\pi _c, \end{aligned}$$

whence, using (3.35) and (3.20), $f_R(s) \ge 1/[1+R_0(1-s)]$. Now *z* is the smallest solution in [0, 1] of $f_R(s)=s$. Thus $z \ge \min (R_0^{-1},1)$, the smallest solution in [0, 1] of $1/[1+R_0(1-s)]=s$.

Turning to ${\tilde{\tau }}_{\epsilon }={\tilde{\tau }}_{\epsilon }(R_0, C, \varvec{\pi })$, note from (3.16), (3.2) and (3.3) that, for $0 \le y \le 1$,

$$\begin{aligned} {\tilde{g}}(y)=\frac{1}{y}\sum _{c=2}^{\infty }p_C(c) c\left[ 1-(1-y\pi _c)^{c-1}\right] \le \sum _{c=2}^{\infty } p_C(c) c(c-1)\pi _c = \frac{R_0}{\lambda }. \end{aligned}$$

Now ${\tilde{y}}(t)$ follows the ODE (3.18), with $\gamma =1$, and ${\tilde{\tau }}_{\epsilon }=\inf \{t>0:{\tilde{y}}(t)=0\}$. Hence, by comparison with (4.4), ${\tilde{y}}(t) \le {\bar{y}}(t)$ $(0 \le t \le {\tilde{\tau }}_{\epsilon })$, where ${\bar{y}}(t)$ is given by (4.5). Thus, ${\tilde{\tau }}_{\epsilon } \le \tau _{\epsilon }^*(R_0)$.

(b) Let ${\tilde{y}}(t)$ $(t \ge 0)$ follow (3.18), with $\gamma =1$ and $\epsilon =0$. Note that for $y>0$, ${\tilde{g}}(y)< y^{-1}\sum _{c=2}^{\infty } p_C(c)c$. Hence, for $0<t< {\tilde{\tau }}_0$,

$$\begin{aligned} \dfrac{d{\tilde{y}}}{dt}=\lambda (1-{\tilde{y}}- t) {\tilde{g}}({\tilde{y}})-1 < \frac{\lambda \sum _{c=2}^{\infty } p_C(c)c}{{\tilde{y}}}-1. \end{aligned}$$

Setting $\gamma =1$ in the expression (3.20) for $R_0$ yields $\lambda =a(C, \varvec{\pi })R_0/\mu _C$. Therefore, ${\tilde{y}}(t) \le a(C, \varvec{\pi }) R_0$ for all $t \in [0, {\tilde{\tau }}]$, since $\dfrac{d{\tilde{y}}}{dt} <0$ if ${\tilde{y}}> a(C, \varvec{\pi }) R_0$.

There exists $t_0 \in (0, {\tilde{\tau }}_0)$ such that ${\tilde{x}}(t_0)=R_0^{-1}$. (This is intuitively clear since otherwise the epidemic will always be supercritical. A proof that ${\tilde{x}}(t) \ge R_0^{-1}$ for all $t \in [0, {\tilde{\tau }}_0]$ leads to a contradiction is given near (7.36) in Sect. 7.4.) Recall that ${\tilde{x}}(t)+{\tilde{y}}(t)=1-t$ for all $t \ge 0$ and that ${\tilde{x}}(t)$ is monotonically decreasing. It follows that ${\tilde{\tau }}_0=1-{\tilde{x}}({\tilde{\tau }}_0) > 1-{\tilde{x}}(t_0)=1-R_0^{-1}$, proving the first inequality in (4.6).

Let ${\tilde{x}}_0=R_0^{-1}$ and ${\tilde{y}}_0={\tilde{y}}(t_0)$. Let $({\check{x}}(t),{\check{y}}(t))$ follow the time-transformed deterministic epidemic obtained by setting $\gamma =1$ in (3.15) but with initial condition $({\check{x}}(0),{\check{y}}(0))=({\tilde{x}}_0, {\tilde{y}}_0)$. Then ${\check{x}}(t)+{\check{y}}(t)={\tilde{x}}_0+{\tilde{y}}_0-t$ $(t \ge 0)$, so ${\check{y}}(t)$ satsifies

$$\begin{aligned} \dfrac{d{\check{y}}}{dt}=\lambda ({\tilde{x}}_0+{\tilde{y}}_0-{\check{y}}- t) {\tilde{g}}({\check{y}})-1, \qquad {\check{y}}(0)={\tilde{y}}_0. \end{aligned}$$

Let ${\check{\tau }}\; (={\check{\tau }}({\tilde{y}}_0))=\inf \{t>0:{\check{y}}(t)=0\}$. Note that ${\tilde{\tau }}_0=t_0+{\check{\tau }}$. Further, ${\tilde{x}}(t_0)+{\tilde{y}}(t_0)=1-t_0$, so

4.8 $$\begin{aligned} {\tilde{\tau }}_0=1-{\tilde{x}}(t_0)-{\tilde{y}}(t_0)+{\check{\tau }}= 1-R_0^{-1}-{\tilde{y}}_0+{\check{\tau }}. \end{aligned}$$

It follows from the proof of part (a) that ${\check{y}}(t) \le {\bar{y}}(t)$, for $0 \le t \le {\check{\tau }}$, where ${\bar{y}}(t)$ satisfies

$$\begin{aligned} \dfrac{d{\bar{y}}}{dt}=R_0 ({\tilde{x}}_0+{\tilde{y}}_0-{\bar{y}}- t)-1, \qquad {\bar{y}}(0)={\tilde{y}}_0, \end{aligned}$$

with solution $${\bar{y}}(t)={\tilde{y}}_0+R_0^{-1}(1-\textrm{e}^{-R_0 t})-t$$.

Thus, ${\check{\tau }}({\tilde{y}}_0) \le f_{R_0}^{-1}({\tilde{y}}_0)$. Further, it is easily verified that ${\check{\tau }}({\tilde{y}}_0)-{\tilde{y}}_0$ is increasing in ${\tilde{y}}_0$. Recall that ${\tilde{y}}_0 \le a(C, \varvec{\pi }) R_0$. Hence, ${\check{\tau }}({\tilde{y}}_0)-{\tilde{y}}_0 \le f_{R_0}^{-1}(a(C, \varvec{\pi })R_0)-a(C, \varvec{\pi }) R_0$, which on substituting into (4.8) yields the second inequality in (4.6).

Turning to (4.7), note that $f_{{\tilde{Z}}}(s) > 0$ for $s \ge 0$, since $R_0>1$ precludes $\textrm{P}({\tilde{Z}}=0)=1$. Hence, recalling $\lambda =a(C, \varvec{\pi })R_0/\mu _C$, it follows from (3.35) that $f_R(s) > 1/[1+a(C, \varvec{\pi })R_0]$ for $s \in [0,1]$. The lower bound for *z* in (4.7) follows, since *z* is the smallest solution in [0, 1] of $f_R(s)=s$. The upper bound is immediate as $R_0>1$. $\square$

Theorem 4.2(a) shows that if $R_0>1$ is held fixed, the standard homogeneously mixing epidemic ($C \sim \text {Const}(2)$) always provides an upper bound for the model with mixing events. Theorem 4.2(b) provides useful bounds when mixing group sizes can be large. Suppose that $C \sim \text {Const}(c)$ and $\pi _c=\pi \in (0, 1]$. Then $a(C, \varvec{\pi })=\frac{1}{(c-1)\pi }$. It follows that when *c* is large, the probability of a major outbreak is small (0 in the limit $c \rightarrow \infty$) and if one occurs its size is slightly larger than $1-\frac{1}{R_0}$ (its limit as $c \rightarrow \infty$). Thus for large *c*, in the event of a major outbreak, the epidemic effectively stops spreading once it becomes critical. More generally, these conclusions hold if $a(C, \varvec{\pi })$ is small.

The proof of Theorem 4.2(b) also has implications for the duration of an epidemic. The duration of the standard homogeneously mixing epidemic is studied in  Barbour (1975). For epidemics with few initial infectives, a major outbreak can be split into three phases: an initial phase, the main body of the epidemic and a final phase, during which the cumulative number infected increases to $\epsilon _1 n$, $(\tau -\epsilon _2)n$ and $\tau n$, respectively, where $\epsilon _1$ and $\epsilon _2$ are both small. For large *n*, the durations of the initial and final phases are both of exact order $\Theta (\log n)$, and the duration of the middle phase is of exact order $\Theta (1)$, more precisely the time for *x*(*t*) to decrease from $1-\epsilon _1$ to $\tau +\epsilon _2$ in the limiting deterministic model. A similar decomposition holds for the model with mixing events. The durations of the initial and final phases of a major outbreak, during which the epidemic can be approximated by a supercritical and a subcritical branching process, respectively, are again of exact order $\Theta (\log n)$. The proof of Theorem 4.2(b) implies that $y(t) \le a(C, \varvec{\pi })R_0$ throughout the middle phase, so the duration of the middle phase is very long if $a(C, \varvec{\pi })$ is small, and tends to infinity as $c \rightarrow \infty$ in the above case when all mixing events have size *c*.

## Special cases

### Logarithmic event distribution

Suppose that *C* follows a logarithmic distribution with parameter $\alpha \in (0,1)$, i.e.

$$\begin{aligned} p_C (c) = \kappa _\alpha \frac{(1-\alpha )^c}{c} \qquad (c=2,3,\ldots ), \end{aligned}$$

where $\kappa _\alpha = [ - \log (\alpha ) - (1-\alpha )]^{-1}$. Thus $\mu _C=\frac{\kappa _\alpha (1-\alpha )^2}{\alpha }$, $\textrm{E}[C(C-1)]=\kappa _\alpha \left( \frac{1-\alpha }{\alpha }\right) ^2$, so $R_0=\frac{\lambda \pi \kappa _\alpha (1-\alpha )^2}{\gamma \alpha ^2}$. Further,

5.1 $$\begin{aligned} f_{{\hat{C}}-2}(s)=\left( \frac{\alpha }{1-(1-\alpha )s}\right) ^2 \qquad (0 \le s \le 1). \end{aligned}$$

Hence, using (4.2),

$$\begin{aligned} U(y)=\frac{R_0}{\pi } \int _{1-\pi y}^1 \left( \frac{\alpha }{1-(1-\alpha )x}\right) ^2\,\textrm{d}x=\frac{R_0 \alpha y}{\alpha +(1-\alpha )\pi y}, \end{aligned}$$

and application of Proposition 4.1(a)(ii) yields that, when $R_0>1$,

$$\begin{aligned} z=\frac{\alpha +(1-\alpha )\pi }{\alpha R_0+(1-\alpha )\pi }. \end{aligned}$$

Now $f_{{\hat{C}}-2}(s)$ is increasing in $\alpha$ for any $s \in [0, 1]$ and strictly increasing for $s \in [0, 1)$. It follows using Theorem 4.1(b)(i) that if $R_0>1$ and $\pi$ are held fixed then both the probability and final size of a major outbreak are increasing in $\alpha$. Moreover, these epidemic properties tend to those of the standard SIR model as $\alpha \rightarrow 1$. Note also that, in the notation of Theorem 4.2(b), $a(C, \varvec{\pi })=\frac{\alpha }{\pi }$. Thus, by that theorem, the fraction of the population infected by a major outbreak tends to $1-R_0^{-1}$ as $\alpha \downarrow 0$.

### Geometric event distribution

Suppose that *C* follows a Geometric distribution with parameter $\alpha \in (0, 1]$, conditioned to be strictly greater than 1, so

$$\begin{aligned} p_C (c) = (1-\alpha )^{c-2} \alpha \qquad (c=2,3,\ldots ). \end{aligned}$$

Thus $\mu _C=1+\frac{1}{\alpha }$. Further, $\textrm{E}[C(C-1)]=\frac{2}{\alpha ^2}$, so $R_0=\frac{2\lambda \pi }{\gamma \alpha ^2}$. Also,

5.2 $$\begin{aligned} f_{{\hat{C}}-2}(s)=\left( \frac{\alpha }{1-(1-\alpha )s}\right) ^3 \qquad (0 \le s \le 1). \end{aligned}$$

Hence, using (4.2),

$$\begin{aligned} U(y)=\frac{R_0}{\pi } \int _{1-\pi y}^1 \left( \frac{\alpha }{1-(1-\alpha )x}\right) ^3\,\textrm{d}x=\frac{R_0 \alpha y[2\alpha +(1-\alpha )\pi y]}{2[\alpha +(1-\alpha )\pi y]^2}. \end{aligned}$$

Let *w* be the largest solution in [0, 1] of $(1-y)U(y)=y$. A little algebra shows that, if $R_0>1$, then *w* satisfies a quadratic equation and application of Proposition 4.1 yields

$$\begin{aligned} z=\frac{(1-\alpha )\pi [\alpha R_0+4(\alpha +(1-\alpha )\pi )]+2\alpha ^2 R_0-\alpha \rho (\alpha , R_0, \pi )}{2(1-\alpha )\pi [\alpha R_0+2\pi (1-\alpha )]}, \end{aligned}$$

where $$\rho (\alpha , R_0, \pi )=\sqrt{[2\alpha +(1-\alpha )\pi ]^2R_0^2+8(1-\alpha )\pi [\alpha +(1-\alpha )\pi ]R_0}$$.

The probability-generating function $f_{{\hat{C}}-2}(s)$ is increasing in $\alpha$ for any $s \in [0, 1]$, so the behaviour of the epidemic properties with $\alpha$, noted in Sect. 5.1 for logarithmic event distributions, hold also for geometric event distributions. Note that, in an obvious notation, for fixed $\alpha \in (0,1)$, $f_{{\hat{C}}-2}^\textrm{geom}(s) < f_{{\hat{C}}-2}^\textrm{log}(s)$ $(0 \le s <1)$. Hence, by Theorem 4.1(b)(i), if $R_0>1$ and $\alpha$ are held fixed, then both the probability and final size of a major outbreak are greater when the sizes of mixing events follow a logarithmic distribution than when they follow a geometric distribution.

## Numerical illustrations

In this section we present numerical results which demonstrate the usefulness of our CLTs for finite population size *n* and illustrate the mixing group size distribution, mean mixing group size and $\pi$ affect model properties. The recovery rate $\gamma =1$ throughout this section.

**Fig. 2:**
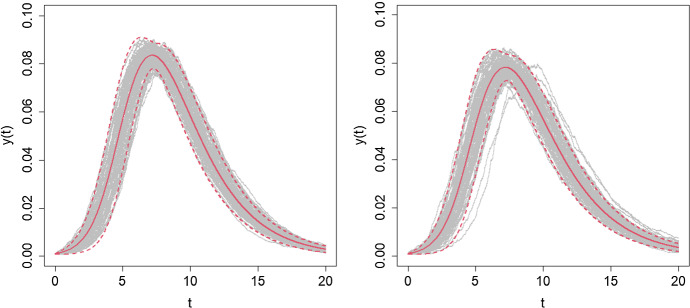
Trajectories of the proportion of the population infectived, *y*(*t*), over the time interval [0, 20] in populations of size $n=100,000$ starting with 100 infectives ($\epsilon = 0.001$) with $R_0 =2$ and $\pi =1$ for 100 simulations (grey lines), along with the deterministic solution (solid red lines) and $95\%$ equal-tailed probability intervals (dashed red lines). Left: Logarithmic distribution with $\alpha = 0.2$, $\mu _C = 3.95$. Right: Geometric distribution with $\alpha = 0.25$, $\mu _C = 5$. See text for further details (color figure online)

In Fig. 2, we consider epidemics in a population of size $n=100,000$ with examples of a logarithmic and a geometric mixing event distribution with $R_0 =2$. For the two mixing event distributions, we plot the trajectories of infectives for the time interval [0, 20], given by the solution of the ODE (3.1) scaled by the population size *n*, together with $95\%$ equal-tailed probability intervals obtained using the functional CLT, Theorem 3.2 with $\epsilon =0.001$ (100 initial infectives). Superimposed on each mixing event distribution plot are 100 simulations of the epidemic with 100 initial infectives on the interval [0, 20]. There is very good agreement between the simulations and the asymptotic limits with excellent coverage of the probability intervals by the simulated trajectories. We note that for the geometric mixing event distribution two of the simulations experience a delay in becoming established and consequently their trajectories have peak later. This is a consequence of variability in the early stages of an epidemic before the number of infectives has increased to a level at which the deterministic approximation fully takes hold.

**Table 1:** Mean proportion infected and scaled variance for epidemics with mixing groups $C=3$. See text for further details

$R_0$	$\pi$	Cut off	Mean proportion				Scaled variance			
			$n=10^4$	$n=10^5$	$n=10^6$	$n=\infty$	$n=10^4$	$n=10^5$	$n=10^6$	$n=\infty$
1.2	0.2	1250	0.3112	0.3121	0.3123	0.3123	16.2035	14.9072	14.4556	14.7263
1.5	0.2	2000	0.5786	0.5787	0.5788	0.5788	3.546	3.5934	3.5044	3.5517
2.0	0.2	2000	0.7912	0.7913	0.7913	0.7913	0.8705	0.8814	0.8835	0.8808
3.0	0.2	2000	0.9364	0.9364	0.9364	0.9364	0.1387	0.1404	0.1377	0.1394
1.2	0.5	1000	0.3090	0.3099	0.3102	0.3102	17.2785	16.3980	15.7946	16.1613
1.5	0.5	1000	0.5726	0.5729	0.5729	0.5729	3.9736	3.8318	3.8020	3.8313
2.0	0.5	1000	0.7831	0.7832	0.7832	0.7832	0.9574	0.9506	0.9680	0.9559
3.0	0.5	1000	0.9301	0.9301	0.9301	0.9301	0.1586	0.1625	0.1607	0.1589
1.2	1.0	5000$^*$	0.3053	0.3066	0.3068	0.3068	21.6961	18.8937	18.7667	18.4861
1.5	1.0	2000	0.5628	0.5636	0.5635	0.5635	4.3695	4.2914	4.3409	4.3022
2.0	1.0	2000	0.7700	0.7701	0.7701	0.7701	1.0876	1.0882	1.1113	1.0900
3.0	1.0	2000	0.9193	0.9193	0.9193	0.9193	0.1925	0.1941	0.1966	0.1956

$^{*}$ For $n=10^4$ the cut-off is at 1000 infectives

**Fig. 3:**
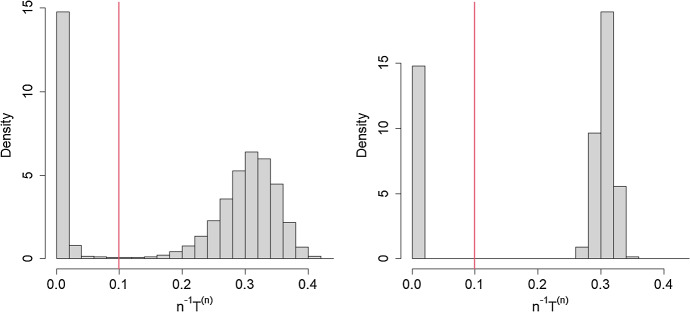
Proportion of population infected by epidemics. Histograms of 10,000 simulations of epidemics in populations of size $n=10,000$ (left panel) and $n=100,000$ (right panel), with $R_0=1.2, \pi =1$ and mixing group size $C=3$. All simulated epidemics had 10 initial infectives

In Table 1, we show how as $n \rightarrow \infty$ the mean proportion ($n^{-1}\textrm{E}[T^{(n)}]$) and scaled variance ($n^{-1}\text {var}(T^{(n)})$) of the final size of a major outbreak approach their asymptotic limits obtained from Theorem 3.5. For fixed size mixing groups of size $C=3$, 10,000 major outbreaks, each initiated by a single infective, were simulated for each of the following combinations of $R_0$ $(=1.2, 1.5, 2.0, 3.0)$, $\pi$ $(= 0.2, 0.5, 1.0)$ and *n* $(=10^4, 10^5, 10^6)$. For each set of parameter values, a cut-off for what constitutes a major outbreak for finite *n* is required. Figure 3 shows histograms of the proportion of the population ever infected in 10,000 simulated epidemics for each of $n=10,000$ and $n=100,000$, when $R_0=1.2, \pi =1$ and $C=3$, with each simulated epidemic being initiated by 10 infectives. (We initiated the epidemics with 10 rather than one infective, since with one initial infective very small minor outbreaks dominate the histograms.) There is a clear distinction between minor and major outbreaks when $n=100,000$ but when $n=10,000$ there is no clear distinction and the choice of cut-off is more arbitrary. The vertical red lines show the cut-off used to produce the corresponding entries in Table 1. The cut-offs for other choices of parameter values and *n* were determined by similar examination of histograms. More generally, the lack of distinction between minor and major outbreaks becomes more pronounced as the size of mixing groups increases for $R_0$ and $\pi$ close to 1. Note from Table 1 that there is excellent agreement between the asymptotic and finite *n* values for the mean proportion of the population infected by a major outbreak. There is also good agreement for the variances for all choices of *n* unless $R_0$ is close to one, in which case *n* needs to be larger for the asymptotic results to provide a good approximation.

**Fig. 4:**
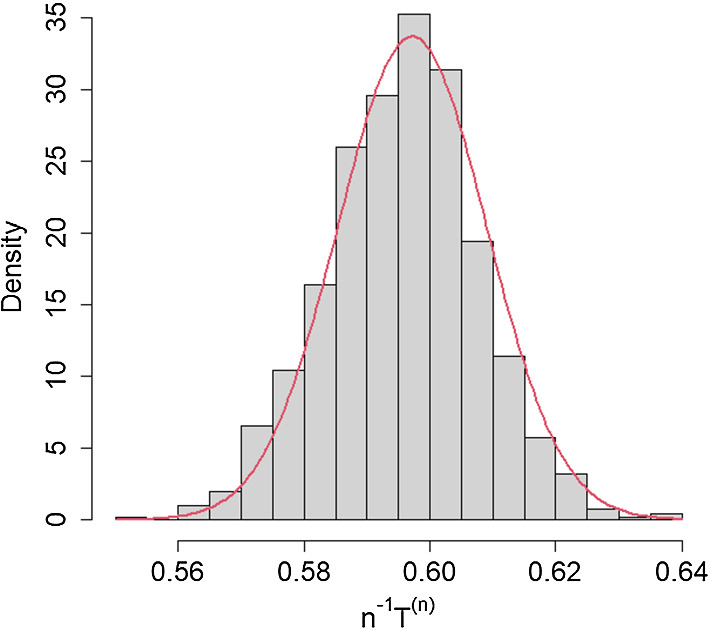
Proportion of the population infected in a major outbreak, $n^{-1}T^{(n)}$. Histogram for 1,000 simulations in population of size $n=10^5$ with $R_0 =2$, $\pi =0.50$ and a logarithmic mixing event distribution with $\alpha = 0.05$ ($\mu _C = 8.82$). Superimposed is the probability density function of $N(0.5972,0.01184^2)$, the approximating normal distribution. See text for further details

In Fig. 4, we illustrate the CLT for the final size using 1,000 simulations of major outbreaks, with a cut-off of $=1,000$, initiated by a single infective in a population of size $n=100,000$, with $R_0 =2$, $\pi =0.50$ and a logarithmic mixing event distribution with $\alpha = 0.05$ ($\mu _C = 8.82$). The asymptotic values of $n^{-1}\textrm{E}[T^{(n)}]$ and $n^{-1}\text {var}(T^{(n)})$ are ${\tilde{\tau }}_0=0.5972$ and $\sigma _T^2(0)=14.0274$, respectively, giving an approximate normal distribution for the proportion infected as $N (0.5972,0.01184^2)$. The superimposed normal approximation closely matches the histogram of the proportions infected in a major outbreak.

**Fig. 5:**
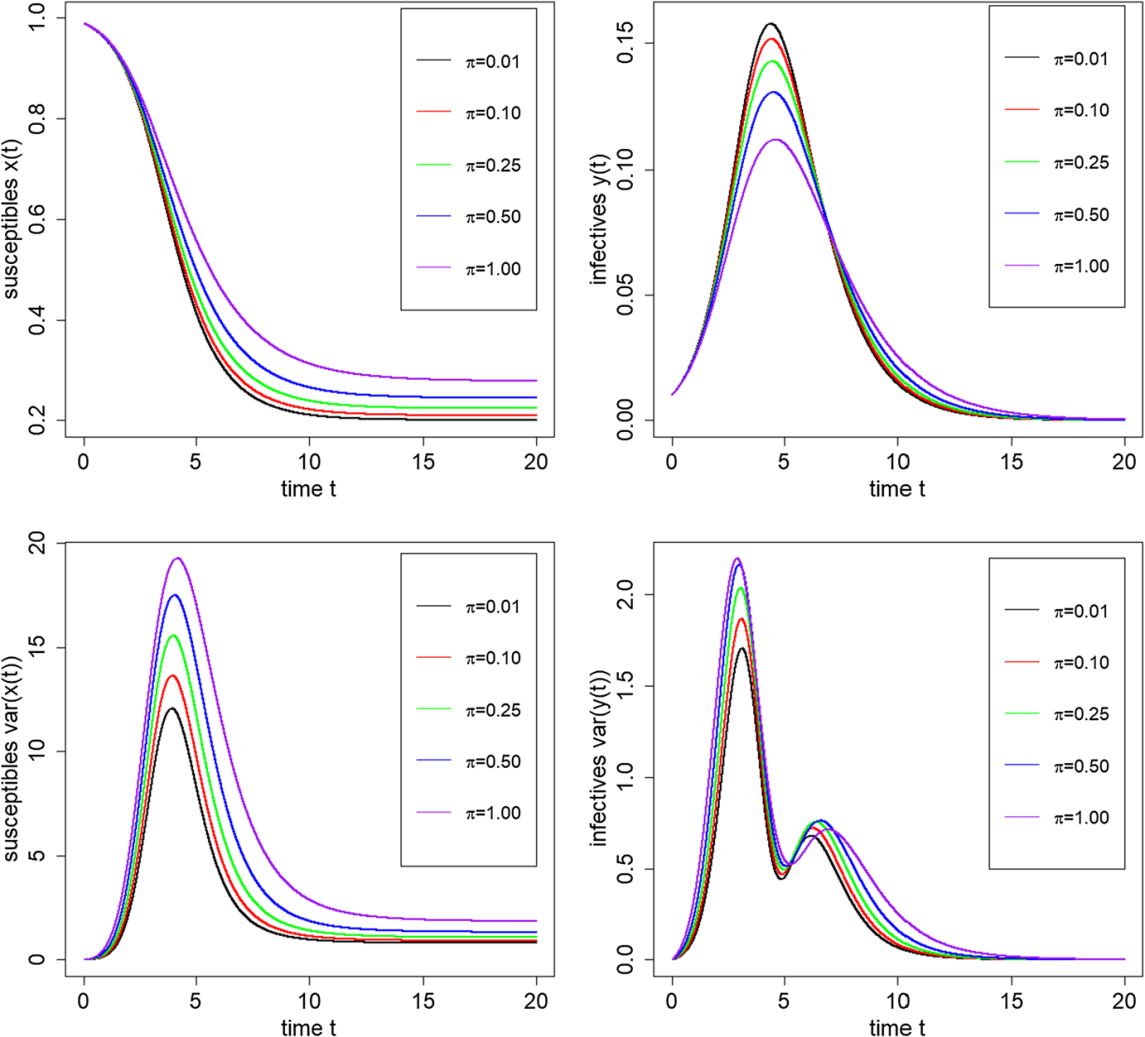
Trajectories of mean proportions and scaled variances of the numbers of susceptibles *x*(*t*) and infectives *y*(*t*) over the time interval [0, 20], for varying $\pi = 0.01, 0.1, 0.25, 0.5, 1.0$ with $R_0 =2$, a logarithmic mixing event distribution with $\alpha =0.35$ ($\mu _C=3.02$) and $\epsilon =0.01$

**Fig. 6:**
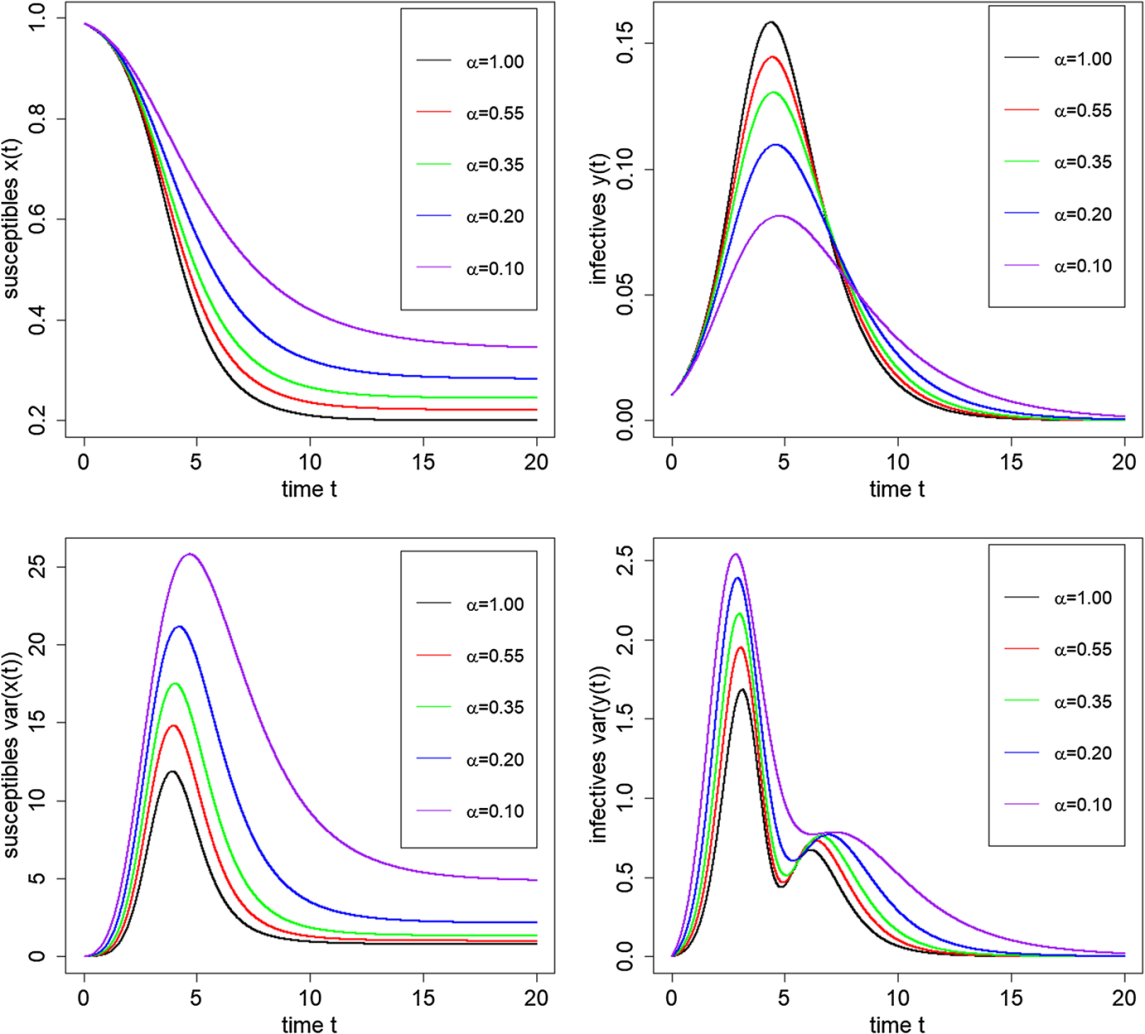
Trajectories of mean proportions and scaled variances of the numbers of susceptibles *x*(*t*) and infectives *y*(*t*) over the time interval [0, 20], for logarithmic mixing event distributions with varying $\alpha = 1, 0.55, 0.35, 0.20, 0.10$ ($\mu _C = 2.00, 2.49, 3.02, 3.95, 5.78$), with $R_0 =2$, $\pi =0.5$ and $\epsilon =0.01$

In Figs. 5 and 6 we show the trajectories of the mean proportion and scaled variance of susceptibles and infectives over the time interval [0, 20] for logarithmic mixing event distributions with $R_0 = 2$ and $\epsilon =0.01$. In Fig. 5, we vary $\pi$ between 0.01 and 1 keeping $\alpha = 0.35$ fixed, so $\mu _C = 3.02$. In Fig. 6, we fix $\pi =0.5$ and vary $\alpha$ between 0.1 and 1, corresponding to mean mixing event sizes ranging from 5.78 down to 2 ($C {\mathop {\longrightarrow }\limits ^{\textrm{D}}}\textrm{Const}(2)$ as $\alpha \uparrow 1$). We observe that as the final size decreases the epidemic has a smaller peak and heavier tail corresponding to a longer duration of the epidemic. This is accompanied by greater variability in the number of susceptibles and infectives over time. Further, if we vary $\epsilon$, the initial proportion infected, say to $\epsilon =0.001$, the effect on the mean trajectories is small, approximately corresponding to a time-shift in the trajectory, but the variance trajectories change significantly, with an increase in magnitude by a factor of between 5 and 10 at the peak and also a larger distinction between the two modes in the variance.

**Fig. 7:**
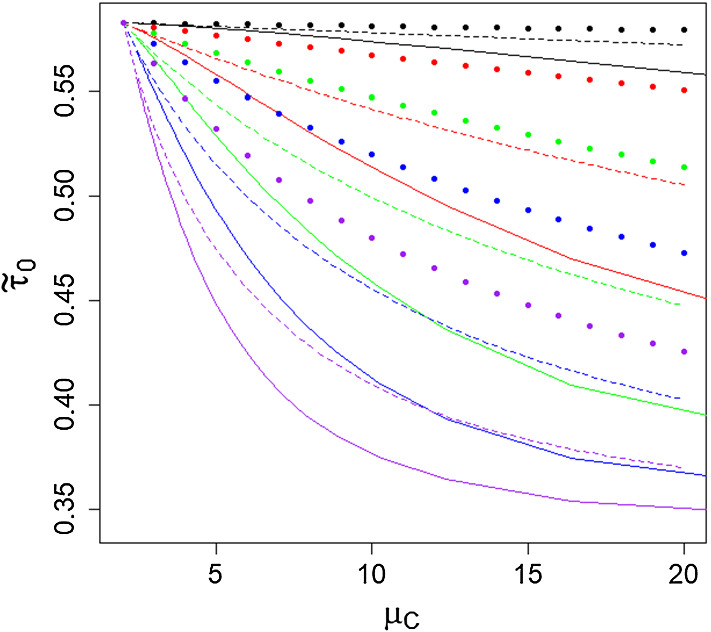
Proportion of individuals infected, ${\tilde{\tau }}_0$, against the mean mixing event size, $\mu _C$, for $R_0=1.5$. Solid line – logarithmic distribution, dashed line – geometric distribution and points – fixed size distribution. $\pi =0.01$ (black), 0.10 (red), 0.25 (green), 0.50 (blue) and 1.00 (purple) (color figure online)

In Fig. 7, we demonstrate how the final size (asymptotic mean proportion infected) of the epidemic, ${\tilde{\tau }}_0$, varies with mean mixing event size, $\mu _C$, mixing event distribution (logarithmic, geometric and fixed size) and constant $\pi$ for a fixed $R_0 = 1.5$. We observe that for all mixing event distributions and choice of $\pi$, the final size agrees for $\mu _C=2$ (all events of size 2) and ${\tilde{\tau }}_0$ decreases as $\mu _C$ increases. The decrease in ${\tilde{\tau }}_0$ as $\mu _C$ increases is, as we would expect, more marked the larger $\pi$ is. We also note that the logarithmic distribution has the fastest drop-off, followed by the geometric distribution, with the fixed group size experiencing the slowest reduction as the mean event size grows. We observe that for the logarithmic distribution the final size ${\tilde{\tau }}_0 <0.35$ for $\mu _C = 20$, approaching the lower bound of $1-1/R_0 = 0.3333$.

## Proofs

### Introduction

We prove these theorems by using the theory of density dependent population processes (see eg. Ethier and Kurtz (1986), Chapter 11, and  Pollett (1990)). In Sect. 7.2, we explain how our model fits into that framework and state the general LLN and functional CLT that we use (Theorems 7.1 and 7.2, respectively). In Sect. 7.3, we derive the key limiting drift and variance/covariance functions for application of those theorems and prove that the conditions of these theorems are satisfied in their application to the temporal behaviour of epidemics with many initial infectives (Theorems 3.1 and 3.2). In Sect. 7.4, we consider the CLTs for the final size of epidemics (Theorems 3.3 and 3.5).

### Density dependent formulation

The process $\{(S^{(n)}(t),I^{(n)}(t))\}=\{(S^{(n)}(t),I^{(n)}(t)): t \ge 0\}$ is a continuous-time Markov chain with state space $E^{(n)}=\{(s,i) \in {\mathbb {Z}}^2:s \ge 0, i \ge 0, s+i \le n\}$ and possible jumps $\Delta ^{(n)}=\{(0,-1), (-1,1), (-2,2),\dots ,(-(n-1),n-1)\}$. The transition intensity for the recovery jump $(0, -1)$ is

$$\begin{aligned} q^{(n)}((s,i),(s,i-1))=\gamma i, \end{aligned}$$

and the transition intensities for infection jumps can be given as follows. For $c=2,3,\dots , n$, consider a mixing event of size *c* that occurs when $(S^{(n)}(t),I^{(n)}(t))=(s,i)$, where $s,i>0$. For $j=0,1,\dots , c-1$, let $p^{(n)}_c(j\vert s,i)$ be the probability that this mixing event yields *j* new infectives. Then, for $k=1,2,\dots , \min (c,i)$, the transition intensity for the jump $(-k,k)$ is

$$\begin{aligned} q^{(n)}((s,i),(s-k,i+k))=n \lambda \sum _{c=2}^n \textrm{P}(C^{(n)}=c)p^{(n)}_c(k\vert s,i) =n \lambda \sum _{c=2}^n p^{(n)}_C(c)p^{(n)}_c(k\vert s,i). \end{aligned}$$

Let ${\varvec{l}}_0=(0,-1)$ and ${\varvec{l}}_j=(-j,j)$ $(j=1,2,\dots )$. The transition intensities take the form

7.1 $$\begin{aligned} q^{(n)}((s,i),(s,i)+{\varvec{l}})=n \beta ^{(n)}_{{\varvec{l}}}\left( \frac{s}{n},\frac{i}{n}\right) , \end{aligned}$$

for nonnegative continuous functions $\beta ^{(n)}_{{\varvec{l}}}:E \rightarrow {\mathbb {R}}$ $({\varvec{l}}\in \Delta ^{(n)})$, where $E=\{(x,y)\in {\mathbb {R}}^2: x \ge 0, y \ge 0, x+y \le 1\}$. Let $\Delta =\{(0,-1), (-1,1), (-2,2), \dots \}$ and suppose further that

$$\begin{aligned} \lim _{n \rightarrow \infty } \beta ^{(n)}_{{\varvec{l}}}(x,y)=\beta _{{\varvec{l}}}(x,y) \qquad ({\varvec{l}}\in \Delta , (x,y) \in E), \end{aligned}$$

where $\beta ^{(n)}_{{\varvec{l}}}: E \rightarrow {\mathbb {R}}$ $({\varvec{l}}\in \Delta )$ are continuous.

Define the drift functions ${\varvec{F}}^{(n)}:E \rightarrow {\mathbb {R}}^2$ and ${\varvec{F}}: E \rightarrow {\mathbb {R}}^2$ by

7.2 $$\begin{aligned} {\varvec{F}}^{(n)}(x,y)=\sum _{{\varvec{l}}\in \Delta ^{(n)}} {\varvec{l}}\beta ^{(n)}_{{\varvec{l}}}(x,y) \qquad \text{ and }\qquad {\varvec{F}}(x,y)=\sum _{{\varvec{l}}\in \Delta } {\varvec{l}}\beta _{{\varvec{l}}}(x,y), \end{aligned}$$

and suppose that ${\varvec{F}}^{(n)}$ converges pointwise to ${\varvec{F}}$ on *E* as $n \rightarrow \infty$. The family of processes $\{(S^{(n)}(t),I^{(n)}(t))\}$ $(n=1,2,\dots )$ is asymptotically density dependent as defined in  Pollett (1990), Definition 3.1. (The theory in  Pollett (1990) requires the set *E* to be open and we define *E* accordingly in applications.)

Write ${\varvec{F}}(x,y)=[F_1(x,y), F_2(x,y)]$, let $(x_0, y_0) \in E$ and $((x(t), y(t)): t\ge 0)$ be the solution of the system of ODEs

7.3 $$\begin{aligned} \dfrac{dx}{dt}=F_1(x,y), \qquad \dfrac{dy}{dt}=F_2(x,y), \qquad (x(0), y(0))=(x_0, y_0). \end{aligned}$$

The following theorem follows from  Pollett (1990), Theorem 3.1.

#### Theorem 7.1

Suppose that ${\varvec{F}}$ is Lipschitz continuous on *E*,

7.4 $$\begin{aligned} \lim _{n \rightarrow \infty } n^{-1}(S^{(n)}(0),I^{(n)}(0))=(x_0, y_0), \end{aligned}$$

and

7.5 $$\begin{aligned} \lim _{n \rightarrow \infty } \sup _{(x,y) \in E}\vert {\varvec{F}}^{(n)}(x,y)-{\varvec{F}}(x,y)\vert =0. \end{aligned}$$

Then, for any $t_0>0$,

7.6 $$\begin{aligned} \sup _{0 \le t \le t_0} \left| n^{-1}(S^{(n)}(t), I^{(n)}(t))-(x(t), y(t))\right| {\mathop {\longrightarrow }\limits ^{\textrm{p}}}0 \quad \text{ as } n \rightarrow \infty . \end{aligned}$$

For $(x,y) \in E$ define the infinitesimal variance/covariance matrices

7.7 $$\begin{aligned} {\varvec{G}}^{(n)}(x,y)=\sum _{{\varvec{l}}\in \Delta ^{(n)}} {\varvec{l}}^{\top } {\varvec{l}}\beta ^{(n)}_{{\varvec{l}}}(x,y) \qquad \text{ and }\qquad {\varvec{G}}(x,y)=\sum _{{\varvec{l}}\in \Delta } {\varvec{l}}^{\top } {\varvec{l}}\beta _{{\varvec{l}}}(x,y), \end{aligned}$$

and let

$$\begin{aligned} \varvec{\partial }{\varvec{F}}(x,y)=\begin{bmatrix} \dfrac{\partial F_1}{\partial x}(x,y) &{} \dfrac{\partial F_1}{\partial y}(x,y) \\ \dfrac{\partial F_2}{\partial x}(x,y) &{} \dfrac{\partial F_2}{\partial y}(x,y) \end{bmatrix}. \end{aligned}$$

For $0 \le u \le t \le \infty$, let $\varvec{\Phi }(t,u)$ be the solution of the matrix ODE

7.8 $$\begin{aligned} \dfrac{\partial }{\partial t}\varvec{\Phi }(t,u)=\varvec{\partial }{\varvec{F}}(x(t), y(t))\varvec{\Phi }(t,u), \quad \varvec{\Phi }(u,u)={\varvec{I}}. \end{aligned}$$

The following theorem follows from  Pollett (1990), Theorem 3.2. The covariance function for the limiting Gaussian process is taken from Ethier and Kurtz (1986), Chapter 11, equations (2.19) and (2.21).

#### Theorem 7.2

Suppose that ${\varvec{G}}$ is bounded and uniformly continuous on *E*,

7.9 $$\begin{aligned} \lim _{n \rightarrow \infty } \sup _{(x,y) \in E} \left| {\varvec{G}}^{(n)}(x,y)-{\varvec{G}}(x,y)\right| =0, \end{aligned}$$

${\varvec{F}}$ has uniformly continuous first partial derivatives on *E*,

7.10 $$\begin{aligned} \lim _{n \rightarrow \infty }\sqrt{n}\sup _{(x,y) \in E}\left| {\varvec{F}}^{(n)}(x,y)-{\varvec{F}}(x,y)\right| =0 \end{aligned}$$

and

7.11 $$\begin{aligned} \lim _{n \rightarrow \infty } \sqrt{n}\left[ n^{-1}(S^{(n)}(0), I^{(n)}(0))-(x(0), y(0))\right] ={\varvec{v}}_0, \end{aligned}$$

where ${\varvec{v}}_0$ is constant. Then

7.12 $$\begin{aligned} \left\{ \sqrt{n}\left[ n^{-1}(S^{(n)}(t), I^{(n)}(t))-(x(t), y(t))\right] \right\} \Rightarrow \{{\varvec{V}}(t): t \ge 0\} \quad \text{ as } n \rightarrow \infty ,\nonumber \\ \end{aligned}$$

where $\{{\varvec{V}}(t)\}$ is a zero-mean Gaussian process with ${\varvec{V}}(0)={\varvec{v}}_0$ and

7.13 $$\begin{aligned} \textrm{cov}\left( {\varvec{V}}(t_1), {\varvec{V}}(t_2)\right) =\int _0^{\min (t_1,t_2)}\varvec{\Phi }(t_1,u) {\varvec{G}}(x(u), y(u))\varvec{\Phi }(t_2,u) ^\top \,\textrm{d}u \qquad (t_1,t_2 \ge 0).\nonumber \\ \end{aligned}$$

### Proofs of Theorems 3.1 and 3.2

We first determine the limiting drift and variance/covariance functions ${\varvec{F}}$ and ${\varvec{G}}$. Recall that in the epidemic in a population of size *n*, the individuals involved in a given mixing event of size *c* are chosen by sampling *c* individuals uniformly at random from the population *w*ithout replacement. In the limit as $n \rightarrow \infty$ this converges to a corresponding sampling *w*ith replacement. For $(x,y) \in E$ and $c=2,3,\dots$, let $\mu _c(x,y)=\textrm{E}[Z]$ and $\mu _{c,2}(x,y)=\textrm{E}[Z^2]$, where *Z* is the number of new infectives created in a mixing event of size *c* in which each of the *c* individuals is independently susceptible, infective or recovered with probabilities *x*, *y* and $1-x-y$, respectively.

#### Lemma 7.1

For $$(x,y) \in E$$ and $$c=2,3,\dots $$,

7.14 $$\begin{aligned} \mu _c(x,y)=cx \left[ 1-(1-y\pi _c)^{c-1}\right] \end{aligned}$$

and

7.15 $$\begin{aligned} \mu _{c,2}(x,y)&=c x \left[ 1-(1-y\pi _c)^{c-1}\right] +c(c-1)x^2\left\{ 1-2(1-y\pi _c)^{c-2}\right. \nonumber \\&\quad \left. +[1-y\pi _c(2-\pi _c)]^{c-2}\right\} . \end{aligned}$$

#### Proof

Label the individuals in the mixing event $1,2,\dots ,c$. For $i=1,2,\dots ,c$, let $\chi _i=1$ if individual *i* is infected at the mixing event and $\chi _i=0$ otherwise, so $Z=\chi _1+\chi _2+\dots +\chi _c$ and by exchangeability $\mu _c(x,y)=c\textrm{P}(\chi _1=1)$. Now $\chi _1=1$ if and only if individual 1 is susceptible, which occurs with probability *x*, and at least one of the individuals $2,3,\dots ,c$ makes infectious contact with individual 1 at the event, which occurs with probability $1-(1-y\pi _c)^{c-1}$. Hence, $P(\chi _1=1)=x [1-(1-y\pi _c)^{c-1}]$ and (7.14) follows.

By exchangeability, $\mu _{c,2}(x,y)=c \textrm{P}(\chi _1=1)+c(c-1) \textrm{P}(\chi _1=1, \chi _2=1)$. Now $\chi _1=\chi _2=1$ if and only if individuals 1 and 2 are both susceptible, which occurs with probability $x^2$, and each of individuals 1 and 2 receive infectious contact from at least one of individuals $3,4,\dots ,c$. For $i=1,2$, let $\eta _i=1$ if at least one of individuals $3,4,\dots ,c$ make infectious contact with individual *i* and $\eta _i=0$ otherwise. The probability that both 1 and 2 avoid infectious contact from individual 3 is $1-y+y(1-\pi _c)^2$, so $\textrm{P}(\eta _1=\eta _2=0)=(1-y+y(1-\pi _c)^2)^{c-2}$. Now $\textrm{P}(\eta _1=0)= (1-y \pi _c)^{c-2}$, so

$$\begin{aligned} \textrm{P}(\eta _1=1,\eta _2=1)= & {} 1-\textrm{P}(\{\eta _1=0\}\cup \{\eta _2=0\})\\= & {} 1-2(1-y\pi _c)^{c-2}+[1-y\pi _c(2-\pi _c)]^{c-2}, \end{aligned}$$

and (7.15) follows. $$\square $$

Note that $\mu _c(x,y)=x g_c(y)$ and $\mu _{c,2}(x,y)=h_c(x,y)$, where the functions $g_c$ and $h_c$ are defined at (3.3) and (3.8), respectively. Since $C^{(n)}{\mathop {\longrightarrow }\limits ^{\textrm{p}}}C$ as $n \rightarrow \infty$, where $\textrm{P}(C=c)=p_C(c)$ $(c=2,3,\dots )$, it follows from their definitions at  (7.2) and (7.7) that ${\varvec{F}}$ and ${\varvec{G}}$ are given by (3.4) and (3.6).

We show via a sequence of lemmas that the conditions of Theorems 7.1 and 7.2 are satisfied. For $i,j=0,1,\dots$, let

$$\begin{aligned} S(i,j)=\sum _{c=2}^{\infty } c^i \pi _c^j p_C(c). \end{aligned}$$

#### Lemma 7.2

- Suppose that $\textrm{E}[C]< \infty$ and $S(2,1)< \infty$. Then ${\varvec{F}}$ is Lipschitz continuous on *E*.
- Suppose that $\textrm{E}[C]< \infty$, $S(2,1)< \infty$ and $S(3,2)< \infty$. Then ${\varvec{F}}$ has uniformly continuous first partial derivatives on *E*.
- Suppose that $\textrm{E}[C^2]< \infty$ and $S(3,1)< \infty$. Then ${\varvec{G}}$ is bounded and uniformly continuous on *E*.

#### Proof

(a) First note that $g_c'(y)=c(c-1)\pi _c(1-y\pi _c)^{c-2}$, so $0 \le g_c'(y)\le c(c-1)\pi _c$ $(0 \le y \le 1)$. Thus $\sum _{c=2}^{\infty }p_C(c) g_c'(y)$ is uniformly convergent on [0, 1], since $S(2,1)< \infty$, so by  Bagley (1959), Theorem 1, $g'(y)=\sum _{c=2}^{\infty }\pi _c g_c'(y)$ for $y \in [0,1]$. Further $0 \le g'(y) \le S(2,1)$ for all $y \in [0,1]$. It follows that the partial derivatives of ${\varvec{F}}$ are bounded on *E*, so ${\varvec{F}}$ is Lipschitz continuous on *E*.

(b) Note that $g_c''(y)=-c(c-1)(c-2)\pi _c^2(1-y\pi _c)^{c-3}$, so arguing as above $g''(y)=\sum _{c=2}^{\infty }\pi _c g_c''(y)$ and $-S(3,2) \le g''(y) \le 0$ $(y \in [0,1])$. Hence $g'$ is uniformly continuous on [0, 1] and part (b) follows.

(c) Note that $0 \le h(x,y) \le \textrm{E}[C^2]$ for all $(x,y) \in E$, so ${\varvec{G}}$ is bounded on *E*. Omitting the details, elementary calculus yields that for all $(x,y) \in E$,

$$\begin{aligned} 0 \le \dfrac{\partial h_c}{\partial x}(x,y) \le c(2c-1) \quad \text{ and }\quad 0 \le \dfrac{\partial h_c}{\partial y}(x,y) \le c(c-1)(2c-3)\pi _c. \end{aligned}$$

Since $\textrm{E}[C^2]< \infty$ and $S(3,1)< \infty$, it follows that the partial derivatives of *h* exist and are bounded on *E*, so *h* is uniformly continuous on *E*, whence so is ${\varvec{G}}$. $\square$

We consider now the convergence of ${\varvec{F}}^{(n)}$ and ${\varvec{G}}^{(n)}$ to ${\varvec{F}}$ and ${\varvec{G}}$ as $n \rightarrow \infty$. Let $E_n=n^{-1}E^{(n)}$ and note from (7.1) that the transition intensities of $\{(S^{(n)}(t),I^{(n)}(t))\}$ require only the values of $\beta ^{(n)}_{{\varvec{l}}}(x,y)$ for $(x,y) \in E_n$. Thus in defining the functions $\beta ^{(n)}_{{\varvec{l}}}$ $({\varvec{l}}\in \Delta ^{(n)})$ we are free to interpolate continuously between the points of $E_n$ rather than using the functional form arising from the appropriate hypergeometric distribution. Recall $\mu _c(x,y)$ and $\mu _{c,2}(x,y)$ defined just before Lemma 7.1. For $c=2,3,\dots$, $n=c,c+1,\dots$ and $(x,y) \in E_n$ consider a mixing event of size *c* in a population of size *n* that contains $s=nx$ susceptibles and $i=ny$ infectives. Let $Z_n$ be the number of new infectives created at this mixing event, $\mu ^{(n)}_c(x,y)=\textrm{E}[Z_n]$ and $\mu ^{(n)}_{c,2}(x,y)=\textrm{E}[Z_n^2]$.

#### Lemma 7.3

- For $c=2,3,\dots$, $n=c,c+1,\dots$ and all $(x,y) \in E_n$,
  7.16 $$\begin{aligned} \vert \mu ^{(n)}_c(x,y)-\mu _c(x,y)\vert \le \frac{c^2(c-1)}{2n} \quad \text{ and }\quad \vert \mu ^{(n)}_{c,2}(x,y)-\mu _{c,2}(x,y)\vert \le \frac{c^3(c-1)}{2n}.\nonumber \\ \end{aligned}$$
- The functions $\beta ^{(n)}_{{\varvec{l}}}$ can be defined using continuous interpolation between the points of $E_n$, so that for $c=2,3,\dots$, $n=c,c+1,\dots$ and all $(x,y) \in E$,
  7.17 $$\begin{aligned} \vert \mu ^{(n)}_c(x,y)-\mu _c(x,y)\vert \le \frac{c^3}{n} \qquad \text{ and }\qquad \vert \mu ^{(n)}_{c,2}(x,y)-\mu _{c,2}(x,y)\vert \le \frac{c^4}{n}.\nonumber \\ \end{aligned}$$

#### Proof

(a) Fix $c \in \{2,3,\dots \}$ and $n \in \{c,c+1,\dots \}$, and for given $(x,y) \in E_n$ define coupled realisations of $Z_n$ and *Z* as follows. Label the individuals in the population $1,2,\dots ,n$, so *nx* of these individuals are susceptible, *ny* are infective and $n(1-x-y)$ are recovered, and let $\zeta ^{(n)}_1, \zeta ^{(n)}_2, \dots$ be i.i.d. discrete uniform random variables on $\{1,2,\dots ,n\}$. For *Z* the disease status of the *c* individuals in the mixing event are given by those of $\zeta ^{(n)}_1, \zeta ^{(n)}_2, \dots , \zeta ^{(n)}_c$. For $Z_n$ they are given by the disease status of the first *c* distinct individuals in $\zeta ^{(n)}_1, \zeta ^{(n)}_2, \dots$. Let $D_n$ be the event that $\zeta ^{(n)}_1, \zeta ^{(n)}_2, \dots , \zeta ^{(n)}_c$ are distinct. If $D_n$ occurs the spread of infection is coupled so that $Z_n=Z$. The precise construction of $(Z_n,Z)$ when $D_n$ does not occur is not needed for our argument. For an event, *D* say, $1_D$ denotes its indicator function. Now

7.18 $$\begin{aligned} \textrm{E}[Z_n]=\textrm{E}[Z_n 1_{D_n}]+\textrm{E}[Z_n 1_{D_n^c}]\qquad \text{ and }\qquad \textrm{E}[Z]=\textrm{E}[Z 1_{D_n}]+\textrm{E}[Z 1_{D_n^c}].\nonumber \\ \end{aligned}$$

Further, $\textrm{E}[Z_n 1_{D_n}]=\textrm{E}[Z 1_{D_n}]$ by construction and $0 \le Z, Z_n \le c$, so

$$\begin{aligned} \vert \textrm{E}[Z_n]-\textrm{E}[Z]\vert = \vert \textrm{E}[Z_n 1_{D_n^c}]-\textrm{E}[Z 1_{D_n^c}] \vert \le c \textrm{P}(D_n^c). \end{aligned}$$

Also,

7.19 $$\begin{aligned} \textrm{P}(D_n^c)=1-\prod _{i=1}^{c-1}\left( 1-\frac{i}{n}\right) \le \sum _{i=1}^{c-1}\frac{i}{n}= \frac{c(c-1)}{2n}. \end{aligned}$$

The first inequality in (7.16) follows. The second one is proved similarly using $0 \le Z^2, Z_n^2 \le c^2$.

(b) Fix *n*. Since the two inequalities in part (a) hold for all $(x,y) \in E_n$, and $\beta _{{\varvec{l}}}$ $({\varvec{l}}\in \Delta )$, $\mu _c$ and $\mu _{c,2}$ are continuous on *E*, $\beta ^{(n)}_{{\varvec{l}}}$ $({\varvec{l}}\in \Delta ^{(n)})$ may be interpolated continuously between the points of $E_n$ so that the two inequalities in (7.17) hold for all $(x,y) \in E$. $\square$

For $n=2,3,\dots$ and $(x,y) \in E$, let

$$\begin{aligned} {\bar{g}}^{(n)}(x,y)=\sum _{c=2}^n p^{(n)}_C(c)\mu ^{(n)}_c(x,y) \qquad \text{ and }\qquad h^{(n)}(x,y)=\sum _{c=2}^n p^{(n)}_C(c) \mu ^{(n)}_{c,2}(x,y). \end{aligned}$$

Observe, using (7.2) and (7.7), that

7.20 $$\begin{aligned} {\varvec{F}}^{(n)}(x,y)= & {} (-\lambda {\bar{g}}^{(n)}(x,y), \lambda {\bar{g}}^{(n)}(x,y)-\gamma y) \text{ and } {\varvec{G}}^{(n)}(x,y)\nonumber \\= & {} \begin{bmatrix} \lambda h^{(n)}(x,y) &{} -\lambda h^{(n)}(x,y) \\ -\lambda h^{(n)}(x,y)&{} \lambda h^{(n)}(x,y)+\gamma y \end{bmatrix}. \end{aligned}$$

#### Lemma 7.4

Suppose that $C^{(n)}{\mathop {\longrightarrow }\limits ^{\textrm{D}}}C$ as $n \rightarrow \infty$.

- If $\textrm{E}[C^{(n)}] \rightarrow \textrm{E}[C]$ as $n \rightarrow \infty$ and $\textrm{E}[C^3]< \infty$ then
  7.21 $$\begin{aligned} \lim _{n \rightarrow \infty } \sup _{(x,y) \in E} \left| {\bar{g}}^{(n)}(x,y)-xg(y)\right| =0. \end{aligned}$$
- If $\textrm{E}[(C^{(n)})^2] \rightarrow \textrm{E}[C^2]$ as $n \rightarrow \infty$ and $\textrm{E}[C^4]< \infty$ then
  7.22 $$\begin{aligned} \lim _{n \rightarrow \infty } \sup _{(x,y) \in E} \left| h^{(n)}(x,y)-h(x,y)\right| =0. \end{aligned}$$
- If $\lim _{n \rightarrow \infty } \sqrt{n}\sum _{c=2}^{\infty }c\vert p^{(n)}_C(c)-p_C(c)\vert =0$ and $\textrm{E}[C^3]<\infty$ then
  7.23 $$\begin{aligned} \lim _{n \rightarrow \infty } \sqrt{n}\sup _{(x,y) \in E} \left| {\bar{g}}^{(n)}(x,y)-xg(y)\right| =0. \end{aligned}$$

#### Proof

(a) Note that $p^{(n)}_C(c)=0$ for $c>n$. Thus,

7.24 $$\begin{aligned} \sup _{(x,y) \in E} \left| {\bar{g}}^{(n)}(x,y)-xg(y)\right|&=\sup _{(x,y) \in E} \left| \sum _{c=2}^{\infty }p^{(n)}_C(c)\mu ^{(n)}_c(x,y)-\sum _{c=2}^{\infty }p_C(c)\mu _c(x,y)\right| \nonumber \\&\le \sup _{(x,y) \in E}\left| \sum _{c=2}^{\infty }\left[ p^{(n)}_C(c)\mu ^{(n)}_c(x,y)-p_C(c)\mu ^{(n)}_c(x,y)\right] \right| \nonumber \\&\quad +\sup _{(x,y) \in E}\left| \sum _{c=2}^{\infty }\left[ p_C(c)\mu ^{(n)}_c(x,y)-p_C(c)\mu _c(x,y)\right] \right| \nonumber \\&\le \sup _{(x,y) \in E}\sum _{c=2}^{\infty }\mu ^{(n)}_c(x,y)\left| p^{(n)}_C(c)-p_C(c)\right| \nonumber \\ {}&\quad +\sup _{(x,y) \in E}\sum _{c=2}^{\infty }p_C(c)\left| \mu ^{(n)}_c(x,y)-\mu _c(x,y)\right| \nonumber \\&\le \sum _{c=2}^{\infty } c \left| p^{(n)}_C(c)-p_C(c)\right| +\sum _{c=2}^{\infty } p_C(c)\frac{c^3}{n}, \end{aligned}$$

where at the final step we have used $\mu ^{(n)}_c(x,y) \le c$ for all $(x,y) \in E$ in the first inequality and Lemma 7.3(b) in the second inequality. Now $\lim _{n \rightarrow \infty } cp^{(n)}_C(c)=c p_C(c)$ for each $c=2,3,\dots$, so $\lim _{n \rightarrow \infty }\sum _{c=2}^{\infty } c \left| p^{(n)}_C(c)-p_C(c)\right| =0$ by Scheffé’s lemma, since $\textrm{E}[C^{(n)}] \rightarrow \textrm{E}[C]$ as $n \rightarrow \infty$. Also, $\sum _{c=2}^{\infty } p_C(c)\frac{c^3}{n} \rightarrow 0$ as $n \rightarrow \infty$, since $\textrm{E}[C^3]< \infty$, and (7.21) follows.

(b) This is analogous to the proof of part (a).

(c) Arguing as in the proof of part (a) yields (c.f. (7.24))

7.25 $$\begin{aligned} \sqrt{n}\sup _{(x,y) \in E} \left| {\bar{g}}^{(n)}(x,y)-xg(y)\right| \le \sqrt{n}\sum _{c=2}^{\infty } c \left| p^{(n)}_C(c)-p_C(c)\right| +\sqrt{n}\sum _{c=2}^{\infty } p_C(c)\frac{c^3}{n}.\nonumber \\ \end{aligned}$$

As $n \rightarrow \infty$, the first term on the right-hand side of (7.25) tends to 0 by assumption and the second term tends to 0 as $\textrm{E}[C^3]< \infty$, so (7.23) follows. $\square$

We now use the above lemmas to show that the conditions of Theorems 7.1 and 7.2 are satisfied. Note from the proofs of those theorems that the conditions on ${\varvec{F}}, {\varvec{G}}, {\varvec{F}}^{(n)}$ and ${\varvec{G}}^{(n)}$ only need to be satisfied in an open neighbourhood of the limiting trajectory $((x(t), y(t)): t\ge 0)$; see eg. the first line of the proof of Ethier and Kurtz (1986), Theorem 11.2.1. For any $t_0>0$, the trajectory $((x(t), y(t)): 0 \le t \le t_0)$ is contained in the interior $E^{{\textsf{o}}}$ of *E*, since $\epsilon >0$, so we may use $E^{{\textsf{o}}}$ in the definition of $\{(S^{(n)}(t),I^{(n)}(t))\}$ $(n=1,2,\dots )$ as an asymptotically density dependent family.

To prove Theorem 3.1, note that ${\varvec{F}}$ is Lipschitz continuous on *E* by Lemma 7.2(a), since $S(2,1)<\textrm{E}[C^2]<\infty$, and that condition (7.4) is satisfied by assumption. Further, recalling (7.20), Lemma 7.4(a) shows that condition (7.5) is satisfied. Thus all the conditions of Theorem 7.1 are satisfied and Theorem 3.1 follows.

Turning to Theorem 3.2, condition (7.9) is satisfied using Lemma 7.4(b) and (7.20), and ${\varvec{G}}$ is bounded and uniformly continuous on *E* by Lemma 7.2(c), since $S(3,1)<\textrm{E}[C^3]$. Condition (7.10) is satisfied using Lemma 7.4(c) and (7.20), and ${\varvec{F}}$ has uniformly continuous first partial derivatives on *E* by Lemma 7.2(b), since $S(2,1)<\textrm{E}[C^2]$ and $S(3,2)<\textrm{E}[C^3]$. Finally, condition (7.11) is satisfied by assumption, so Theorem 3.2 follows.

### Proofs of Theorems 3.3 and 3.5

The final size $T^{(n)}$ of ${\mathscr {E}}^{(n)}$ is given by $T^{(n)}=n-S^{(n)}(\tau ^{(n)})$, where $\tau ^{(n)}=\inf \{t>0:I^{(n)}(t)=0\}$. We study the asymptotic distribution of $T^{(n)}$ via the exit of the process $\{(S^{(n)}(t),I^{(n)}(t))\}$ from $E^{(n)}_+$, the set of states in $E^{(n)}$ with $i>0$. Under the conditions of Theorem 3.3, $\tau ^{(n)}{\mathop {\longrightarrow }\limits ^{\textrm{p}}}\infty$ as $n \rightarrow \infty$, so to obtain a process which exits $E^{(n)}_+$ in finite time in the limit as $n \rightarrow \infty$, we consider $\{({\tilde{S}}^{(n)}(t),{\tilde{I}}^{(n)}(t))\}$, a random time-scale transformation of $\{(S^{(n)}(t),I^{(n)}(t))\}$ in which, at any time $t \ge 0$, the clock is slowed down by a factor $n^{-1}I^{(n)}(t)$. Thus $\{({\tilde{S}}^{(n)}(t),{\tilde{I}}^{(n)}(t))\}$ is a continuous-time Markov chain with transition intensities (cf. (7.1)) satisfying, for $(s,i) \in E^{(n)}_+$ and ${\varvec{l}}\in \Delta ^{(n)}$,

7.26 $$\begin{aligned} {\tilde{q}}^{(n)}((s,i),(s,i)+{\varvec{l}})=\frac{n}{i}q^{(n)}((s,i),(s,i)+{\varvec{l}}) =n{\tilde{\beta }}^{(n)}_{{\varvec{l}}}\left( \frac{s}{n},\frac{i}{n}\right) , \end{aligned}$$

where ${\tilde{\beta }}^{(n)}_{{\varvec{l}}}(x,y)=y^{-1}\beta ^{(n)}_{{\varvec{l}}}(x,y)$ $({\varvec{l}}\in \Delta ^{(n)}, (x,y) \in E^{(n)}_+)$. The use of such random time-scale transformations in epidemic modelling goes back to  Watson (1980).

The jump chains of $\{(S^{(n)}(t),I^{(n)}(t))\}$ and $\{({\tilde{S}}^{(n)}(t),{\tilde{I}}^{(n)}(t))\}$ coincide, so $T^{(n)}{\mathop {=}\limits ^{D}}n-{\tilde{S}}^{(n)}({\tilde{\tau }}^{(n)})$, where ${\tilde{\tau }}^{(n)}=\inf \{t>0:{\tilde{I}}^{(n)}(t)=0\}$. To determine the asymptotic distribution of ${\tilde{S}}^{(n)}({\tilde{\tau }}^{(n)})$ it is fruitful to define the process $\{({\tilde{S}}^{(n)}(t),{\tilde{I}}^{(n)}(t))\}$ beyond time ${\tilde{\tau }}^{(n)}$, which involves allowing ${\tilde{I}}^{(n)}(t)$ to be negative. For $y_0 \in (0,\infty )$, let

$$\begin{aligned} {\tilde{E}}^{(n)}({y_0})=E^{(n)}\cup \{(s,i):0 \le s \le n, -y_0n \le i <0\}, \end{aligned}$$

${\tilde{E}}_n(y_0)=n^{-1} {\tilde{E}}^{(n)}({y_0})$ and ${\tilde{E}}(y_0)=E \cup \left( [0,1] \times [-y_0,0)\right)$. For ${\varvec{l}}\in \Delta$, extend the domain of $\beta _{{\varvec{l}}}$ from *E* to ${\tilde{E}}(y_0)$ by adopting the same functional form. For ${\varvec{l}}\in \Delta ^{(n)}$ and $(x,y) \in {\tilde{E}}^{(n)}({y_0})$, now let $\{{\tilde{S}}^{(n)}(t),{\tilde{I}}^{(n)}(t))\}$ have transition intensities given by

$$\begin{aligned} {\tilde{q}}^{(n)}((s,i),(s,i)+{\varvec{l}})=n{\tilde{\beta }}^{(n)}_{{\varvec{l}}}\left( \frac{s}{n},\frac{i}{n}\right) , \end{aligned}$$

where

7.27 $$\begin{aligned} {\tilde{\beta }}^{(n)}_{{\varvec{l}}}(x,y)= {\left\{ \begin{array}{ll} y^{-1} \beta ^{(n)}_{{\varvec{l}}}(x,y) &{} \text { if } (x,y) \in n^{-1}E^{(n)}_+,\\ y^{-1} \beta _{{\varvec{l}}}(x,y) &{} \text{ otherwise } . \end{array}\right. } \end{aligned}$$

It is possible that $\beta _{{\varvec{l}}}(x,y)<0$ for some (*x*, *y*) with $y<0$. If so, replace ${\varvec{l}}$ by ${\varvec{l}}'=-{\varvec{l}}$ and define $\beta _{{\varvec{l}}'}(x,y)=-\beta _{{\varvec{l}}}(x,y)$ for such (*x*, *y*). This change, which is assumed implicitly in the following, does not effect the resulting infinitesimal drift and variance/covariance functions. In (7.27), $y^{-1}\beta _{{\varvec{l}}}(x,y)$ is defined by continuity when $y=0$. It is immediate that the limit exists for recovery jumps, while for infection jumps the limit exists as for a mixing event to yield new infectives at least one of its members must be an infective. For ${\varvec{l}}\in \Delta$, define ${\tilde{\beta }}_{{\varvec{l}}}$ by ${\tilde{\beta }}_{{\varvec{l}}}(x,y)=y^{-1}\beta _{{\varvec{l}}}(x,y)$ $((x,y) \in {\tilde{E}}(y_0))$.

The limiting infinitesimal drift and variance/covariance of $\{{\tilde{S}}^{(n)}(t),{\tilde{I}}^{(n)}(t))\}$ are

7.28 $$\begin{aligned} \tilde{{\varvec{F}}}(x,y)=(-\lambda x {\tilde{g}}(y), \lambda x {\tilde{g}}(y)-\gamma ) \text{ and } \tilde{{\varvec{G}}}(x,y)=\begin{bmatrix} \lambda {\tilde{h}}(x,y) &{} -\lambda {\tilde{h}}(x,y) \\ -\lambda {\tilde{h}}(x,y)&{} \lambda {\tilde{h}}(x,y)+\gamma \end{bmatrix},\nonumber \\ \end{aligned}$$

where

$$\begin{aligned} {\tilde{g}}(y)=\sum _{c=2}^{\infty } p_C(c){\tilde{g}}_c(y)\qquad \text{ and }\qquad {\tilde{h}}(x,y)= \sum _{c=2}^{\infty } p_C(c){\tilde{h}}_c(x,y), \end{aligned}$$

with, for $$c=2,3,\dots $$,

7.29 $$\begin{aligned} {\tilde{g}}_c(y)= {\left\{ \begin{array}{ll} \frac{c}{y}[1-(1-\pi _cy)^{c-1}] &{} \text { if } y \ne 0,\\ c(c-1)\pi _c &{} \text { if } y=0, \end{array}\right. } \end{aligned}$$

and

7.30 $$\begin{aligned} {\tilde{h}}_c(x,y)= {\left\{ \begin{array}{ll} x{\tilde{g}}_c(y)+\frac{c(c-1)x^2}{y}\left\{ 1-2(1-y\pi _c)^{c-2}+[1-y\pi _c(2-\pi _c)]^{c-2}\right\} &{} \text { if } y \ne 0,\\ c(c-1)\pi _cx+c(c-1)(c-2)\pi _c^2 x^2 &{} \text { if } y=0. \end{array}\right. }\nonumber \\ \end{aligned}$$

The limiting time-transformed deterministic model is given by (3.15). Suppose that $\epsilon \in (0,1)$, as in Theorem 3.3, let ${\tilde{\tau }}_{\epsilon }=\inf \{t>0:{\tilde{y}}(t)=0\}$ and, for $y_0>0$, let ${\tilde{\tau }}_{\epsilon }(y_0)=\inf \{t>0:{\tilde{y}}(t)=-y_0\}$. Note that (3.15) implies that ${\tilde{x}}(t)>0$ for all $t\ge 0$. Hence, using (3.17), ${\tilde{\tau }}_{\epsilon }(y_0)< \infty$ for all $y_0 \ge 0$. Suppose instead that $\epsilon =0$, as in Theorem 3.5. Then, using the expression for $R_0$ at (3.20), it follows from (3.15) that ${\tilde{y}}'(0)=\gamma (R_0-1)$. Let ${\tilde{\tau }}_0=\inf \{t>0:{\tilde{y}}(t)=0\}$, where ${\tilde{\tau }}_0=\infty$ if ${\tilde{y}}<0$ for all $t>0$. If $R_0>1$, as in Theorem 3.5, then ${\tilde{\tau }}_0 \in (0, \infty )$. The above argument shows that ${\tilde{\tau }}_0(y_0)=\inf \{t>0:{\tilde{y}}(t)=-y_0\}$ is finite for all $y_0>0$.

The following theorem is proved via an analogous sequence of lemmas to those used in the proof of Theorem 3.2. The proofs of the lemmas corresponding to Lemmas 7.2 and 7.4 are obvious generalisations, although the details are more involved. The proof of the lemma corresponding to Lemma 7.3 is less immediate. Details may be found in Appendix B in the Supplementary Information.

#### Theorem 7.3

Suppose that $\sqrt{n}(n^{-1}m_n-\epsilon ) \rightarrow \epsilon _0$ and $C^{(n)}{\mathop {\longrightarrow }\limits ^{\textrm{D}}}C$ as $n \rightarrow \infty$, where $\epsilon \ge 0$. Suppose further that conditions (3.27)-(3.30) are satisfied. For $t \ge 0$, let

$$\begin{aligned} \tilde{{\varvec{V}}}^{(n)}(t)=\sqrt{n}\left[ n^{-1}({\tilde{S}}^{(n)}(t), {\tilde{I}}^{(n)}(t))-({\tilde{x}}(t), {\tilde{y}}(t))\right] , \end{aligned}$$

where $({\tilde{x}}(t), {\tilde{y}}(t))$ is given by the solution of (3.15). Then, for any $0< t_0 <{\tilde{\tau }}_{\epsilon }(y_0)$,

7.31 $$\begin{aligned} \{\tilde{{\varvec{V}}}^{(n)}(t): 0 \le t \le t_0\} \Rightarrow \{\tilde{{\varvec{V}}}(t):0 \le t \le t_0\} \quad \text{ as } n \rightarrow \infty , \end{aligned}$$

where $\{\tilde{{\varvec{V}}}(t):0 \le t \le t_0\}$ is a zero-mean Gaussian process with $\tilde{{\varvec{V}}}(0)=(-\epsilon _0,\epsilon _0)$ and variance

7.32 $$\begin{aligned} \tilde{\varvec{\Sigma }}(t)=\textrm{var}\left( \tilde{{\varvec{V}}}(t)\right) = \begin{bmatrix} {\tilde{\sigma }}_{S,\epsilon }^2(t) &{} {\tilde{\sigma }}_{SI, \epsilon }(t) \\ {\tilde{\sigma }}_{SI, \epsilon }(t)&{} {\tilde{\sigma }}_{I,\epsilon }^2(t) \end{bmatrix}, \end{aligned}$$

where $({\tilde{\sigma }}_{S,\epsilon }^2(t),{\tilde{\sigma }}_{SI,\epsilon }(t){\tilde{\sigma }}_{I,\epsilon }^2(t))$ is given by the solution of the system of ODEs (3.21) - (3.23) with initial condition $({\tilde{\sigma }}_{S}^2(0),{\tilde{\sigma }}_{SI}(0), {\tilde{\sigma }}_{I}^2(0))=(0,0,0)$.

Recall that we wish to determine the asymptotic distribution of $T^{(n)}{\mathop {=}\limits ^{D}}n-{\tilde{S}}^{(n)}({\tilde{\tau }}^{(n)})$, where ${\tilde{\tau }}^{(n)}=\inf \{t>0:{\tilde{I}}^{(n)}(t)=0\}$. We now cast the associated boundary crossing problem into the framework of Ethier and Kurtz (1986), Theorem 11.4.1.

Let $\varphi : {\tilde{E}}(y_0)\rightarrow {\mathbb {R}}$ be defined by $\varphi (x,y)=y$. Then ${\tilde{\tau }}^{(n)}=\inf \{t \ge 0:\varphi ({\tilde{S}}^{(n)}(t),{\tilde{I}}^{(n)}(t)) \le 0\}$, ${\tilde{\tau }}_{\epsilon }=\inf \{t \ge 0:\varphi ({\tilde{x}}(t),{\tilde{y}}(t)) \le 0\}$ and, when $\epsilon >0$, $\varphi ({\tilde{x}}(0),{\tilde{y}}(0))>0$. Then, if ${\tilde{\tau }}_{\epsilon }<\infty$ and $\nabla \varphi ({\tilde{x}}({\tilde{\tau }}_{\epsilon }), {\tilde{y}}({\tilde{\tau }}_{\epsilon }))\cdot \tilde{{\varvec{F}}}({\tilde{x}}({\tilde{\tau }}_{\epsilon }),{\tilde{y}}({\tilde{\tau }}_{\epsilon }))<0$, where $\cdot$ denotes inner vector product, it follows from Ethier and Kurtz (1986), Theorem 11.4.1, and Theorem 7.3 that, as $n \rightarrow \infty$,

7.33 $$\begin{aligned} \sqrt{n}&\left( n^{-1}({\tilde{S}}^{(n)}({\tilde{\tau }}^{(n)}),{\tilde{I}}^{(n)}({\tilde{\tau }}^{(n)})))-({\tilde{x}}({\tilde{\tau }}_{\epsilon }), {\tilde{y}}({\tilde{\tau }}_{\epsilon }))\right) \nonumber \\&\qquad \qquad {\mathop {\longrightarrow }\limits ^{\textrm{D}}}{\varvec{V}}({\tilde{\tau }}_{\epsilon }) -\frac{\nabla \varphi ({\tilde{x}}({\tilde{\tau }}_{\epsilon }),{\tilde{y}}({\tilde{\tau }}_{\epsilon }))\cdot \tilde{{\varvec{V}}}({\tilde{\tau }}_{\epsilon })}{\nabla \varphi ({\tilde{x}}({\tilde{\tau }}_{\epsilon }),{\tilde{y}}({\tilde{\tau }}_{\epsilon }))\cdot \tilde{{\varvec{F}}}({\tilde{x}}({\tilde{\tau }}_{\epsilon }),{\tilde{y}}({\tilde{\tau }}_{\epsilon }))}\tilde{{\varvec{F}}}({\tilde{x}}({\tilde{\tau }}_{\epsilon }),{\tilde{y}}({\tilde{\tau }}_{\epsilon })). \end{aligned}$$

Hence, as $$n \rightarrow \infty $$,

7.34 $$\begin{aligned} \sqrt{n}\left( n^{-1}({\tilde{S}}^{(n)}({\tilde{\tau }}^{(n)}),{\tilde{I}}^{(n)}({\tilde{\tau }}^{(n)})))-({\tilde{x}}({\tilde{\tau }}_{\epsilon }), {\tilde{y}}({\tilde{\tau }}_{\epsilon }))\right) {\mathop {\longrightarrow }\limits ^{\textrm{D}}}\textrm{N}({\varvec{0}},{\varvec{B}}\tilde{\varvec{\Sigma }}({\tilde{\tau }}_{\epsilon }) {\varvec{B}}^{\top }),\nonumber \\ \end{aligned}$$

where $\tilde{\varvec{\Sigma }}({\tilde{\tau }}_{\epsilon })$ is given by (7.32) and

$$\begin{aligned} {\varvec{B}}={\varvec{I}}-\frac{\tilde{{\varvec{F}}}({\tilde{x}}({\tilde{\tau }}_{\epsilon }),{\tilde{y}}({\tilde{\tau }}_{\epsilon }))^{\top } \nabla \varphi ({\tilde{x}}({\tilde{\tau }}_{\epsilon }),{\tilde{y}}({\tilde{\tau }}_{\epsilon }))}{\nabla \varphi ({\tilde{x}}({\tilde{\tau }}_{\epsilon }),{\tilde{y}}({\tilde{\tau }}_{\epsilon }))\cdot \tilde{{\varvec{F}}}({\tilde{x}}({\tilde{\tau }}_{\epsilon }),{\tilde{y}}({\tilde{\tau }}_{\epsilon }))}. \end{aligned}$$

Now ${\tilde{y}}({\tilde{\tau }}_{\epsilon })=0$, so using (3.17), ${\tilde{x}}({\tilde{\tau }}_{\epsilon })=1-\gamma {\tilde{\tau }}_{\epsilon }$. Hence, using (7.28),

7.35 $$\begin{aligned} \tilde{{\varvec{F}}}({\tilde{x}}({\tilde{\tau }}_{\epsilon }),{\tilde{y}}({\tilde{\tau }}_{\epsilon }))=(-\gamma R_0(1-\gamma {\tilde{\tau }}_{\epsilon }),\gamma R_0(1-\gamma {\tilde{\tau }}_{\epsilon })-\gamma ), \end{aligned}$$

since, from (3.16) and (3.20), ${\tilde{g}}(0)=\gamma \lambda ^{-1}R_0$. Further, $\nabla \varphi ({\tilde{x}}({\tilde{\tau }}_{\epsilon }),{\tilde{y}}({\tilde{\tau }}_{\epsilon }))=(0,1)$, so

$$\begin{aligned} a= & {} \nabla \varphi ({\tilde{x}}({\tilde{\tau }}_{\epsilon }), {\tilde{y}}({\tilde{\tau }}_{\epsilon }))\cdot \tilde{{\varvec{F}}}({\tilde{x}}({\tilde{\tau }}_{\epsilon }),{\tilde{y}}({\tilde{\tau }}_{\epsilon }))=\gamma (R_0(1-\gamma {\tilde{\tau }}_{\epsilon })-1)\\= & {} \gamma (R_0 {\tilde{x}}({\tilde{\tau }}_{\epsilon })-1). \end{aligned}$$

Now ${\tilde{x}}(t)$ is strictly decreasing with *t*, since ${\tilde{g}}_c(y)>0$ for $y \in (-\infty ,1)$ and $c=2,3,\dots$. Thus $a<0$ if $R_0 {\tilde{x}}(0) \le 1$.

To show that $a<0$ when $R_0 {\tilde{x}}(0) > 1$. Suppose for contradiction that $a \ge 0$. Then, since ${\tilde{x}}(t)$ is decreasing with *t*, it follows from (7.36) that ${\tilde{x}}(t) \ge R_0^{-1}$ for $0 \le t \le {\tilde{\tau }}_{\epsilon }$, so recalling (3.15), ${\tilde{y}}(t) \ge {\hat{y}}(t)$ $(0 \le {\tilde{\tau }}_{\epsilon })$, where ${\hat{y}}(t)$ is the solution of the ODE

7.36 $$\begin{aligned} \dfrac{d{\hat{y}}}{dt}=\lambda R_0^{-1} {\tilde{g}}({\hat{y}})-\gamma ,\qquad {\hat{y}}(0)=\epsilon . \end{aligned}$$

For $$c=2,3,\dots $$ and $$y \in [0,1]$$,

$$\begin{aligned} {\tilde{g}}_c(y) \ge c(c-1)\pi _c-\frac{c(c-1)(c-2) \pi _c^2 y}{2}, \end{aligned}$$

whence

$$\begin{aligned} {\tilde{g}}(y)\ge \sum _{c=2}^{\infty } p_C(c) c(c-1)\pi _c -\frac{y}{2} \sum _{c=2}^{\infty }p_C(c) c(c-1)(c-2)\pi _c^2=\frac{\gamma R_0}{\lambda }-\frac{R_0 b y}{\lambda }, \end{aligned}$$

where

$$\begin{aligned} b=\frac{\lambda }{2R_0}\sum _{c=2}^{\infty } p_C(c) c(c-1)(c-2)\pi _c^2. \end{aligned}$$

Now $b < \infty$, since ${\tilde{S}}_1(3,2,y_0)<\infty$, so using (7.36), ${\hat{y}}(t) \ge {\bar{y}}(t)$ $(0 \le {\tilde{\tau }}_{\epsilon })$ where ${\bar{y}}(t)$ solves

$$\begin{aligned} \dfrac{d{\bar{y}}}{dt}=-b {\bar{y}},\qquad {\bar{y}}(0)=\epsilon , \end{aligned}$$

so ${\bar{y}}(t)=\epsilon \exp (-bt)$ $(t \ge 0)$. Therefore, ${\tilde{y}}({\tilde{\tau }}_{\epsilon }) \ge {\bar{y}}({\tilde{\tau }}_{\epsilon }) >0$, which is a contradiction as ${\tilde{y}}({\tilde{\tau }}_{\epsilon })=0$. Hence, $a<0$, as required. Note that this argument also shows that $a<0$ when $\epsilon =0$ and $R_0>1$, since ${\tilde{y}}'(0)=\gamma (R_0-1)$.

Finally, it follows using (7.35) and (7.36) that

$$\begin{aligned} {\varvec{B}}=\begin{bmatrix} 1 &{} -d_{\epsilon } \\ 0 &{} 0 \end{bmatrix}, \end{aligned}$$

where $d_{\epsilon }$ is given by (3.19). Theorem 3.3 follows from (7.34), since $T^{(n)}{\mathop {=}\limits ^{D}}n-{\tilde{S}}^{(n)}({\tilde{\tau }}^{(n)})$ and ${\tilde{x}}({\tilde{\tau }}_{\epsilon })=1-\gamma {\tilde{\tau }}_{\epsilon }$.

We turn now to the proof of Theorem 3.5, so $R_0>1$ and the initial number of infectives $m_n=m$ for all sufficiently large *n*. Consider the (un-time-transformed) epidemic process $\{(S^{(n)}(t),I^{(n)}(t))\}$ and let $t_n=\inf \{t: S^{(n)}(t) \le n-\log n\}$, where $t_n=\infty$ if $S^{(n)}(t) > n-\log n$ for all $t \ge 0$. Thus $t_n$ is the time when the cumulative number of infectives reaches at least $\log n$, so $t_n< \infty$ if and only if $T^{(n)}\ge \log n$. Suppose $T^{(n)}\ge \log n$. We run the un-time-transformed process up until time $t_n$ and then make the random time-scale transformation described above, with the clock starting again at 0. By the strong Markov property, the latter process is a realisation of $\{({\tilde{S}}^{(n)}(t),{\tilde{I}}^{(n)}(t))\}$ with $({\tilde{S}}^{(n)}(0),{\tilde{I}}^{(n)}(0))=(S^{(n)}(t_n), I^{(n)}(t_n))$. Note that $0 \le n-{\tilde{S}}^{(n)}(0) \le 1+\log n$ and $0 \le {\tilde{I}}^{(n)}(0) \le 1+\log n$, so conditional upon $T^{(n)}\ge \log n$, $\lim _{n \rightarrow \infty } \sqrt{n}\left[ n^{-1}({\tilde{S}}^{(n)}(0), {\tilde{I}}^{(n)}(0))-(1, 0))\right] =(1,0)$, and Theorem 7.3 holds with $\epsilon =0$ and $\epsilon _0=0$.

Recall that ${\tilde{\tau }}^{(n)}=\inf \{t>0:{\tilde{I}}^{(n)}(t)=0\}$ and ${\tilde{\tau }}_0=\inf \{t>0:{\tilde{y}}(t)=0\}$, where ${\tilde{y}}(t)$ is the solution of (3.18) with $\epsilon =0$. Further ${\tilde{\tau }}_0 \in (0, \infty )$ as $R_0>1$. Theorem 7.3 implies that there exists $t_0>{\tilde{\tau }}_0$ such that

7.37 $$\begin{aligned} \sup _{0 \le t \le t_0} \left| n^{-1}({\tilde{S}}^{(n)}(t), {\tilde{I}}^{(n)}(t))-({\tilde{x}}(t), {\tilde{y}}(t))\right| {\mathop {\longrightarrow }\limits ^{\textrm{p}}}0 \quad \text{ as } n \rightarrow \infty , \end{aligned}$$

where $({\tilde{x}}(t), {\tilde{y}}(t))$ is given by the solution of (3.15) with initial condition $({\tilde{x}}(0),{\tilde{y}}(0))=(1,0)$. It follows that $\min ({\tilde{\tau }}^{(n)}, \vert {\tilde{\tau }}^{(n)}-{\tilde{\tau }}_0\vert ) {\mathop {\longrightarrow }\limits ^{\textrm{p}}}0$ as $n \rightarrow \infty$. By Theorem 3.4(b), there exists $\delta >0$ such that

7.38 $$\begin{aligned} \lim _{n \rightarrow \infty } \textrm{P}(T^{(n)}\ge n \delta \vert T^{(n)}\ge \log n)=1. \end{aligned}$$

Let $t_1$ be the unique solution in $(0, \infty )$ of ${\tilde{x}}(t_1)=1-\delta$. Then (7.37) and (7.38) imply that $\textrm{P}({\tilde{\tau }}^{(n)}\le \frac{\delta }{2}\vert T^{(n)}\ge \log n) \rightarrow 0$ as $n \rightarrow \infty$, so

7.39 $$\begin{aligned} \lim _{n \rightarrow \infty }\textrm{P}\left( T^{(n)}=n-{\tilde{S}}^{(n)}({\tilde{\tau }}^{(n)}_1)\vert T^{(n)}\ge \log n\right) =1, \end{aligned}$$

where $${\tilde{\tau }}^{(n)}_1=\inf \{t>\frac{t_1}{2}:{\tilde{I}}^{(n)}(t)=0\}$$.

Now ${\tilde{\tau }}_0=\inf \{t>\frac{t_1}{2}: {\tilde{y}}(t)=0\}$, since ${\tilde{y}}(t)>0$ for all $t \in (0, \frac{t_1}{2}]$. The above proof is easily modified to show that, conditional upon $T^{(n)}\ge \log n$, (7.34) holds with $\epsilon =0$ and ${\tilde{\tau }}^{(n)}$ replaced by ${\tilde{\tau }}^{(n)}_1$. Theorem 3.5 follows using (7.39), since ${\tilde{x}}({\tilde{\tau }}_0)=1-\gamma {\tilde{\tau }}_0$.

## Concluding comments

We have presented a new class of SIR epidemic models in which disease is transmitted via mixing groups, and not just pairwise interactions, together with a branching process approximation for the early phase of an epidemic with few initial infectives and CLTs for both the temporal behaviour of epidemics with many initial infectives and the final outcome of a major outbreak. The standard homogeneously mixing SIR epidemic model is a special case of the mixing group model when mixing groups are necessarily of size 2. If $R_0$ and the recovery rate $\gamma$ are held fixed then the initial exponential growth rate *r* of our model is the same as that of the standard SIR model but both the probability, $1-z$, and final size, $\tau$, of a major outbreak are smaller. We have proved a number of comparison results for the mixing group model. In broad terms, if $R_0$ and $\gamma$ are held fixed, then both $1-z$ and $\tau$ decrease with mixing group size and the duration of an epidemic increases with mixing group size. In the extreme case of very large mixing groups, the final size $\tau$ of a major outbreak can be only fractionally larger than $1-R_0^{-1}$. (Note that $\tau$ is necessarily greater than $1-R_0^{-1}$ since otherwise the epidemic would never become subcritical.)

One limitation of the analysis and results presented is that they assume that the infectious period follows an exponential distribution. The approximating branching process ${\mathscr {B}}$ described in Sect. 3.3 can be extended in the obvious fashion to the case when the infectious period follows an arbitrary but specified distribution. The CLTs presented in this paper can be extended via the method of stages to incorporate an Erlang, and more generally a phase-type, infectious period distribution. An alternative approach is to use the LLN and functional CLT for age and density dependent population processes in  Wang (1975, 1977), though details are likely to be complicated. The phase-type approach is generally easier to implement as it requires just the methodology of density dependent population processes, which is well developed. However, if the infectious period distribution does not belong to the class of phase-type distributions then that approach is only approximate since one needs to approximate the actual infectious period distribution by one which is phase-type. Although any distribution can be approximated arbitrarily closely by a phase-type distribution, the size of the phase space can become infeasible for numerical purposes.

It would be interesting to study other models for transmission within mixing events. One possibility is to make a Greenwood-type assumption: viz. if there are *c* individuals at a mixing event then, provided there is at least one infective at the event, each susceptible present becomes infected independently with probability $\pi _c$. The approximating branching process ${\mathscr {B}}$ is unchanged, as it assumes that all mixing events contain at most one infective. However, the CLTs do change. Omitting the details, the formulae for $g_c(y)$ and $h_c(x,y)$ (see (3.3) and (3.8)) become

$$\begin{aligned} g_c(y)=c \pi _c\left[ 1-(1-y)^{c-1}\right] \end{aligned}$$

and

$$\begin{aligned} h_c(x,y)=c x \pi _c\left[ 1-(1-y)^{c-1}\right] +c(c-1)x^2\pi _c^2\left[ 1-(1-y)\right] ^{c-2}. \end{aligned}$$

Corresponding theorems to Theorems 3.1, 3.2, 3.3 and 3.5 then follow with some changes to their sufficient conditions. Analogous comparison results to Theorems 4.1 and 4.2 hold. Further, if all mixing events are of size *c* and $\pi _c=\min (\zeta /c,1)$, where $\zeta \in (0, \infty )$, then if $R_0>1$ and $\gamma$ are held fixed and $c \rightarrow \infty$, the probability of a minor outbreak given one initial infective, *z*, tends to a limit that is strictly less than 1, while the size of a major outbreak, $\tau$, tends to $1-R_0^{-1}$. The limit of $\tau$ for the corresponding model with Reed-Frost type mixing is strictly greater than $1-R_0^{-1}$.

A few models with non-pairwise transmission are mentioned in the introduction. Other examples include models incorporating synergystic interactions (eg. Ludlam et al. 2012), models in which the usual mass-action law of infection, where the overall force of infection is proportional to the product of the numbers of susceptible (*S*) and infective (*I*) infective individuals, is replaced by some other function of (*S*, *I*) (eg. O’Neill and Wen 2012 and references therein) and in nonparametric inference, such as Knock and Kypraios (2014), where no particular form is assumed for the force of infection. In all of these models new infectives occur one at a time. Billard et al. (1980) analyse an SI model (i.e. one in with no recovery from infection) in which infections occur in batches whose sizes are i.i.d. random variables. However, models in which infection is not driven by pairwise interaction of individuals are relatively rare.

The model was motivated by non-pharmaceutical intervention policies for Covid-19 that placed limits on the size of gatherings outside the home and it would be interesting to use it to explore the effects of such policies. It is straightforward to analyse, at least numerically, the effects of changes in the distribution of the mixing group size random variable *C* on epidemic properties such as $R_0$ and the probability and size of a major outbreak. The LLN and functional CLT in Sect. 3.2 can be extended to allow the distribution of *C* to be time and/or state dependent, thus permitting investigation of corresponding control measures. Another feature that is highly pertinent to Covid-19 spread and control is the role played by households. It would be worthwhile to examine the effects of extending the households model of Ball et al. (1997) so that between-household spread is modelled using mixing groups, rather than by homogeneous mixing. Another promising direction for future work is to introduce heterogeneities, eg. owing to age and/or level of social activity, and study the corresponding multitype model.

## Supplementary Information

Below is the link to the electronic supplementary material.

Supplementary file 1 (pdf 154 KB)

## Data Availability

This manuscript has no associated data.
