# Supplementary material for: An epidemic model with short-lived mixing groups
Source: J Math Biol. 2022 Oct 31;85(6-7):63. doi: 10.1007/s00285-022-01822-3 (PMC9622612; doi:10.1007/s00285-022-01822-3)
Supplement: Supplementary file 1 — (pdf 154 KB) [file 285_2022_1822_MOESM1_ESM.pdf]

Supplementary Information: An epidemic model with mixing groups

Frank Ball[1*] and Peter Neal[2]

[1*]School of Mathematical Sciences, University of Nottingham, University Park, Nottingham, NG7 2RD, UK.
[2]School of Mathematical Sciences, University of Nottingham, University Park, Nottingham, NG7 2RD, Nottingham.

*Corresponding author(s). E-mail(s): frank.ball@nottingham.ac.uk;
Contributing authors: peter.neal@nottingham.ac.uk;

Appendix A Proofs of sufficient conditions for theorems

Suppose that there is a maximum mixing group size, c_* say, so $\mathrm{P}(C^{(n)} > c_*) = 0$ for all n. Then it is immediate that the conditions concerning $C^{(n)}$ and C in all of the theorems are satisfied if

$$\lim_{n\to\infty} \sqrt{n}[p_C^{(n)}(c) - p_C(c)] = 0 \quad (c = 2,3,\dots,c_*),$$

since all sums contain only finitely many terms.

Consider now the case when there is no maximum mixing group size. Suppose that there exists $s_1 > 1$ such that $f_C(s_1) < \infty$. Then $\sum_{c=2}^{\infty} c^3 s_1^{\frac{c}{2}} p_C(c) < \infty$, since $c^3 \leq s_1^{\frac{c}{2}}$ for all sufficiently large c. Noting that $0 \leq \pi_c, \pi_c(2-\pi_c) \leq 1$, it follows that the conditions concerning $\tilde{S}_1(i,j,y_0)$ and $\tilde{S}_2(i,j,y_0)$ in Theorems 3.3 and 3.5 hold with $y_0 = \sqrt{s_1} - 1$. The conditions concerning moments of C (including (3.27)) are satisfied in all of the theorems since C has finite moments of all orders.

The conditions concerning the convergence of $C^{(n)}$ to C are satisfied in all of the theorems if (cf. (3.10) and (3.29))

$$\lim_{n\to\infty} \sqrt{n} \sum_{c=2}^{\infty} c^2 \left| p_C^{(n)}(c) - p_C(c) \right| = 0, \tag{A1}$$

and (cf. (3.28))

$$\lim_{n\to\infty}\sum_{c=2}^{\infty} c^3 p_C^{(n)}(c) = \sum_{c=2}^{\infty} c^3 p_C(c), \tag{A2}$$

since $\mathrm{E}[C^3] < \infty$.

Suppose that $C^{(n)} \stackrel{D}{=} \min(C,n)$ $(n = 2,3,\dots)$. Then

$$\sum_{c=2}^{\infty} c^2 \left| p_C^{(n)}(c) - p_C(c) \right| = n^2 \mathrm{P}(C \geq n+1) + \sum_{c=n+1}^{\infty} c^2 p_C(c).$$

Now $\mathrm{P}(C \geq n+1) \leq \frac{1}{(n+1)^3}\mathrm{E}[C^3]$ by Markov's inequality, so $n^{\frac{5}{2}}\mathrm{P}(C \geq n+1) \to 0$ as $n \to \infty$. Also,

$$\mathrm{E}[C^3] \geq (n+1) \sum_{c=n+1}^{\infty} c^2 p_C(c),$$

so $\sqrt{n}\sum_{c=n+1}^{\infty} c^2 p_C(c) \to 0$ as $n \to \infty$ and (A1) is satisfied. A similar argument using $\mathrm{E}[C^4] < \infty$ shows that (A2) holds.

Suppose instead that $C^{(n)} \stackrel{D}{=} (C|C \leq n)$ $(n = 2,3,\dots)$. Then

$$p_C^{(n)}(c) = \begin{cases} \frac{p_C(c)}{\mathrm{P}(C \leq n)} & \text{if } c = 2,3,\dots,n, \\ 0 & \text{otherwise .} \end{cases}$$

Hence,

$$\sum_{c=2}^{\infty} c^2 \left| p_C^{(n)}(c) - p_C(c) \right| = \frac{1-\mathrm{P}(C \leq n)}{\mathrm{P}(C \leq n)} \sum_{c=2}^{n} c^2 p_C(c) + \sum_{c=n+1}^{\infty} c^2 p_C(c).$$

Now $\mathrm{P}(C \leq n) \to 1$ as $n \to \infty$, so for all sufficiently large n,

$$\frac{1-\mathrm{P}(C \leq n)}{\mathrm{P}(C \leq n)} \sum_{c=2}^{n} c^2 p_C(c) \leq 2\mathrm{P}(C \geq n+1) \sum_{c=2}^{n} c^2 p_C(c) \leq 2n^2 \mathrm{P}(C \geq n+1).$$

The same arguments as used for the case $C^{(n)} \stackrel{D}{=} \min(C,n)$ now show that (A1) and (A2) hold.

We turn now to the case when the infection probabilities π_c $(c = 2,3,\dots)$ satisfy $\pi_c \leq \frac{\zeta}{c}$ for all sufficiently large c, for some $\zeta \in (0,\infty)$. Suppose that $\pi_c \leq \frac{\zeta}{c}$ for all $c \geq c_0$. Then, for fixed $y_0 > 0$,

$$(1+\pi_c(2-\pi_c)y_0)^c \leq \left(1+\frac{2\zeta y_0}{c}\right)^c \leq \mathrm{e}^{2\zeta y_0},$$

for all sufficiently large c. It follows that $\tilde{S}_2(i,i-2,y_0) < \infty$ $(i = 2,3)$ if $\mathrm{E}[C^2] < \infty$. A similar argument shows that $\tilde{S}_1(i,i-1,y_0)$ $(i = 1,2,3)$ are all finite if $\mathrm{E}[C] < \infty$.

The above arguments show that the other conditions of the theorems are satisfied if $\mathrm{E}[C^4] < \infty$, apart from possibly (3.28). Now

$$\begin{aligned}\sum_{c=2}^{\infty} \pi_c c^3 \left| p_C^{(n)}(c) - p_C(c) \right| &= \sum_{c=2}^{c_0-1} \pi_c c^3 \left| p_C^{(n)}(c) - p_C(c) \right| + \sum_{c=c_0}^{\infty} \pi_c c^3 \left| p_C^{(n)}(c) - p_C(c) \right| \\ &\leq \sum_{c=2}^{c_0-1} \pi_c c^3 \left| p_C^{(n)}(c) - p_C(c) \right| + \zeta \sum_{c=c_0}^{\infty} c^2 \left| p_C^{(n)}(c) - p_C(c) \right|. \end{aligned} \tag{A3}$$

As $n \to \infty$, the first sum in (A3) converges to 0, since $\lim_{n\to\infty} p_C^{(n)}(c) = p_C(c)$ $(c = 2,3,\dots,c_0-1)$, and the second sum also converges to 0, since (A1) holds when $\mathrm{E}[C^4] < \infty$. Thus, (3.28) holds. Hence, if $\pi_c \leq \frac{\zeta}{c}$ for all $c \geq c_0$, then $\mathrm{E}[C^4] < \infty$ is sufficient for all the conditions concerning $C^{(n)}$ and C in all of the theorems.

Appendix B Proof of Theorem 7.3

Theorem 7.3 is proved by showing that $\{(\tilde{S}^{(n)}(t), \tilde{I}^{(n)}(t))\}$ $(n = 1,2,\dots)$ satisfies the conditions of Theorem 7.2, with obvious modifications; in particular, E is replaced by $\tilde{E}(y_0)$. The proof parallels that of Theorem 3.2 and involves a corresponding series of lemmas. The details are more involved as the infinitesimal drift and variance/covariance functions take a more complicated form. Also in the proof of the lemma corresponding to Lemma 7.3 (i.e. Lemma B.2 below), that is used to obtain uniform bounds on $|\tilde{\boldsymbol{F}}^{(n)} - \tilde{\boldsymbol{F}}|$ and $|\tilde{\boldsymbol{G}}^{(n)} - \tilde{\boldsymbol{G}}|$, sharper upper bounds on the moments of the number of people infected by a mixing event are required.

Lemma B.1 *Suppose that $C^{(n)} \xrightarrow{\mathrm{D}} C$ as $n \to \infty$ and $y_0 > 0$.*

(a) (i) The series defining $\tilde{g}$ converges on $[-y_0, 1]$ if $\tilde{S}_1(1,0,y_0) < \infty$.
(ii) The drift function $\tilde{\boldsymbol{F}}$ is Lipschitz continuous on $\tilde{E}(y_0)$ if $\tilde{S}_1(i,i-1,y_0) < \infty$ for $i = 1,2$.
(iii) The drift function $\tilde{\boldsymbol{F}}$ has uniformly continuous first partial derivatives on $\tilde{E}(y_0)$ if $\tilde{S}_1(i,i-1,y_0) < \infty$ for $i = 1,2,3$.

(b) The infinitesimal variance/covariance $\tilde{\boldsymbol{G}}$ is bounded and uniformly continuous on $\tilde{E}(y_0)$ if $\tilde{S}_2(i,i-2,y_0) < \infty$ for $i = 2,3$.

Proof (a) Note that

$$\tilde{g}_c(y) = \pi_c c \sum_{i=0}^{c-2} (1 - \pi_c y)^i, \tag{B1}$$

so $\tilde{g}_c$ is positive and decreasing on $[-y_0, 1]$. Part (i) follows since

$$\tilde{g}(-y_0) = \sum_{c=2}^{\infty} p_C(c) \frac{c}{y_0} \left[(1 + \pi_c y_0)^{c-1} - 1 \right] < \infty,$$

as $\tilde{S}_1(1,0,y_0) < \infty$.

For $y \neq 0$,

$$\tilde{g}_c'(y) = \frac{c}{y^2}\left[(c-1)\pi_c y(1-\pi_c y)^{c-2} - 1 + (1-\pi_c y)^{c-1}\right]. \tag{B2}$$

It follows from (B1) that $\tilde{g}_c'(y) \leq 0$ and $\tilde{g}_c''(y) \geq 0$ for $y \in [-y_0, 1]$, so

$$-y_0^{-1}c(c-1)\pi_c(1+\pi_c y_0)^{c-2} - y_0^{-2}c \leq \tilde{g}_c'(y) \leq 0 \qquad (y \in [-y_0,1]),$$

as $(1+\pi_c y_0)^{c-1} > 0$. Suppose that the conditions of part (ii) hold. Then $\sum_{c=2}^{\infty} p_C(c)\tilde{g}_c'$ is uniformly convergent on $[-y_0,1]$. Thus $\tilde{g}'(y) = \sum_{c=2}^{\infty} p_C(c)\tilde{g}_c'(y)$ for $y \in [-y_0,1]$ and moreover $\tilde{g}'$ is bounded on $[-y_0,1]$. Hence, using also part (i), it follows that the partial derivatives of $\tilde{\boldsymbol{F}}$ are bounded on $\tilde{E}(y_0)$, so $\tilde{\boldsymbol{F}}$ is Lipschitz continuous on $\tilde{E}(y_0)$.

Differentiating (B2) yields after a little manipulation that, for $y \neq 0$,

$$\tilde{g}_c''(y) = \frac{c}{y^4}\left\{2y - (1-\pi_c y)^{c-3} y\left(2(1+(c-3)\pi_c y) + [(c-1)(c-4)+2]\pi_c^2 y^2\right)\right\}.$$

Recall that $\tilde{g}_c''(y) \geq 0$ on $[-y_0,1]$. It is seen easily from (B1) that $\tilde{g}_c'''(y) \leq 0$ on $[-y_0,1]$. Thus

$$\tilde{g}_c''(-y_0) \leq \frac{c}{y_0^3}\left\{(1+\pi_c y_0)^{c-3}\left(2 + [(c-1)(c-4)+2]\pi_c^2 y_0^2\right)\right\}.$$

The conditions of part (iii) ensure that $\sum_{c=2}^{\infty} p_C(c)\tilde{g}_c''(-y_0) < \infty$, so arguing as in the proof of part (i) yields that $\tilde{g}''$ exists and is bounded on $[-y_0,1]$. Thus $\tilde{g}'$ is uniformly continuous on $[-y_0,1]$ and part (iii) follows.

(b) Note that, for $c = 2,3,\ldots,$

$$\tilde{h}_c(x,y) = x\tilde{g}_c(y) + c(c-1)x^2[\tilde{g}_{c,1}(y) - \tilde{g}_{c,2}(y)],$$

where

$$\tilde{g}_{c,1}(y) = \frac{1}{y}\left[1 - (1-\pi_c y)^{c-2}\right]$$

and

$$\tilde{g}_{c,2}(y) = \frac{1}{y}\left\{(1-\pi_c y)^{c-2} - [1-\pi_c(2-\pi_c)y]^{c-2}\right\} \tag{B3}$$

$$= \pi_c(1-\pi_c)\sum_{i=0}^{c-3}(1-\pi_c y)^i[1-\pi_c(2-\pi_c)y]^{c-3-i}. \tag{B4}$$

From part (a)(i), $\sum_{c=2}^{\infty} p_C(c)\tilde{g}_c$ is bounded on $[-y_0,1]$ if $\tilde{S}_1(1,0,y_0) < \infty$ and a similar argument shows that $\sum_{c=2}^{\infty} p_C(c)c(c-1)\tilde{g}_{c,1}$ is bounded on $[-y_0,1]$ if $\tilde{S}_1(2,0,y_0) < \infty$.

Using (B4), $\tilde{g}_{c,2}(y) \geq 0$ and $\tilde{g}_{c,2}'(y) \leq 0$ for $y \in [-y_0,1]$, so

$$0 \leq \tilde{g}_{c,2}(y) \leq \frac{1}{y_0}[1+\pi_c(2+\pi_c)y_0]^{c-2} \qquad (y \in [-y_0,1]),$$

and $\sum_{c=2}^{\infty} p_C(c)c(c-1)\tilde{g}_{c,2}$ is bounded on $[-y_0,1]$ if $\tilde{S}_2(2,0,y_0) < \infty$. Noting that $\tilde{S}_1(i,0,y_0) \leq \tilde{S}_2(2,0,y_0)$ $(i=1,2)$, it follows that $\tilde{\boldsymbol{G}}$ and $\dfrac{\partial \tilde{\boldsymbol{G}}}{\partial x}$ are bounded on $\tilde{E}(y_0)$ if $\tilde{S}_2(2,0,y_0) < \infty$.

From the proof of part (a)(ii), $\sum_{c=2}^{\infty} p_C(c)\tilde{g}_c$ is bounded differentiable on $[-y_0,1]$ if $\tilde{S}_1(i,i-1,y_0) < \infty$ $(i=1,2)$. Suppose that $\tilde{S}_1(i,i-2,y_0) < \infty$ $(i=2,3)$. Then a similar argument shows that $\sum_{c=2}^{\infty} p_C(c)c(c-1)\tilde{g}_{c,1}$ is bounded differentiable on $[-y_0,1]$. Thus both $\sum_{c=2}^{\infty} p_C(c)\tilde{g}_c$ and $\sum_{c=2}^{\infty} p_C(c)c(c-1)\tilde{g}_{c,1}$ are bounded differentiable on $[-y_0,1]$, since $\tilde{S}_1(i,i-1,y_0) \leq \tilde{S}_2(i+1,i-1,y_0)$ $(i=1,2)$ and $\tilde{S}_1(i,i-2,y_0) \leq \tilde{S}_2(i,i-2,y_0)$ $(i=2,3)$.

Differentiating (B3),

$$\tilde{g}'_{c,2}(y) = \frac{1}{y^2}\Big\{[1+(c-3)\pi_c(2-\pi_c)y][1+\pi_c(2-\pi_c)y]^{c-3} \\ -[1+(c-3)\pi_c y](1-\pi_c y)^{c-3}\Big\}.$$

Again using (B4), $\tilde{g}''_{c,2}(y) \geq 0$ for $y \in [-y_0, 1]$, so $\sum_{c=2}^{\infty} p_C(c)c(c-1)\tilde{g}'_{c,2}$ is bounded and uniformly convergent on $[-y_0, 1]$ if $\tilde{S}_2(2,0,y_0) < \infty$ and $\tilde{S}_2(3,2,y_0) < \infty$. Thus, under these conditions, $\sum_{c=2}^{\infty} p_C(c)c(c-1)\tilde{g}_{c,2}$ is bounded differentiable on $[-y_0, 1]$. Hence, under the conditions of part (b), $\frac{\partial \tilde{\boldsymbol{G}}}{\partial y}$ is bounded on $\tilde{E}(y_0)$. It then follows that $\tilde{\boldsymbol{G}}$ is uniformly continuous on $\tilde{E}(y_0)$, since both partial derivatives of $\tilde{\boldsymbol{G}}$ are bounded on $\tilde{E}(y_0)$. □

Recall the functions $\mu_c(x,y)$ and $\mu_{c,2}(x,y)$, defined just before Lemma 7.1, and the functions $\mu_c^{(n)}(x,y)$ and $\mu_{c,2}^{(n)}(x,y)$, defined just before Lemma 7.3. For $(x,y) \in E$, let $\tilde{\mu}_c(x,y) = y^{-1}\mu_c(x,y)$ and $\tilde{\mu}_{c,2}(x,y) = y^{-1}\mu_{c,2}(x,y)$. Note that $\tilde{\mu}_c(x,y) = x\tilde{g}_c(x,y)$ and $\tilde{\mu}_{c,2}(x,y) = \tilde{h}_c(x,y)$. Thus, using (7.29) and (7.30), these functions can be extended to have domain $\tilde{E}(y_0)$, although their interpretation in terms of multinomial sampling is valid only for $(x,y) \in E$. For $c = 2,3,\dots, n = c, c+1, \dots$ and $(x,y) \in n^{-1}E_+^{(n)}$, let $\tilde{\mu}_c^{(n)}(x,y) = y^{-1}\mu_c^{(n)}(x,y)$ and $\tilde{\mu}_{c,2}^{(n)}(x,y) = y^{-1}\mu_{c,2}^{(n)}(x,y)$. For $(x,y) \in \tilde{E}(y_0)$, with $y \leq 0$, let $\tilde{\mu}_c^{(n)}(x,y) = \tilde{\mu}_c(x,y)$ and $\tilde{\mu}_{c,2}^{(n)}(x,y) = \tilde{\mu}_{c,2}(x,y)$; cf. (7.27). Moreover, the functions $\tilde{\beta}_{\boldsymbol{l}}^{(n)}$ $(\boldsymbol{l} \in \Delta)$ induce natural extensions of $\tilde{\mu}_c^{(n)}(x,y)$ and $\tilde{\mu}_{c,2}^{(n)}(x,y)$ so that they have domain $\tilde{E}(y_0)$.

Lemma B.2 *(a) For $c = 2,3,\dots$, $n = c, c+1, \dots$ and all $(x,y) \in E_n$ with $y > 0$,*

$$|\tilde{\mu}_c^{(n)}(x,y) - \tilde{\mu}_c(x,y)| \leq \frac{\pi_c c^4}{n} \quad \text{and} \quad |\tilde{\mu}_{c,2}^{(n)}(x,y) - \tilde{\mu}_{c,2}(x,y)| \leq \frac{\pi_c c^5}{n}. \tag{B5}$$

(b) The functions $\tilde{\beta}_{\boldsymbol{l}}^{(n)}$ can be defined using continuous interpolation between the points of E_n, so that for $c = 2,3,\dots$, $n = c, c+1, \dots$ and all $(x,y) \in \tilde{E}(y_0)$,

$$|\tilde{\mu}_c^{(n)}(x,y) - \tilde{\mu}_c(x,y)| \leq \frac{\pi_c c^4}{n} \quad \text{and} \quad |\tilde{\mu}_{c,2}^{(n)}(x,y) - \tilde{\mu}_{c,2}(x,y)| \leq \frac{\pi_c c^5}{n}. \tag{B6}$$

Proof (a) Fix $c \in \{2,3,\dots\}$ and $n \in \{c, c+1, \dots\}$, and for any given $(x,y) \in E_n$, with $y > 0$, define coupled realisations of Z_n and Z as in the proof of Lemma 7.3. It follows from (7.18) and the coupling of Z_n and Z that

$$|\mathrm{E}[Z_n] - \mathrm{E}[Z]| = |\mathrm{E}[Z_n 1_{D_n^c}] - \mathrm{E}[Z 1_{D_n^c}]| = \mathrm{P}(D_n^c)|\mathrm{E}[Z_n|D_n^c] - \mathrm{E}[Z|D_n^c]| \\ \leq \mathrm{P}(D_n^c)\max(\mathrm{E}[Z_n|D_n^c], \mathrm{E}[Z|D_n^c]). \tag{B7}$$

Note that Z_n is constructed using c distinct, exchangeable $(\zeta_i^{(n)})$ individuals, so $Z_n|D_n \stackrel{D}{=} Z_n$, where $\stackrel{D}{=}$ denotes equal in distribution, and $\mathrm{E}[Z_n|D_n^c] = \mathrm{E}[Z_n]$. However, the distribution of Z does depend on D_n; for example, if $c = 2$ and D_n^c occurs, then both individuals in the mixing event have the same disease status and $\mathrm{E}[Z|D_n^c] = 0$.

To obtain a useful upper bound for $y^{-1}\mathrm{E}[Z_n]$, and hence for $y^{-1}\mathrm{E}[Z_n|D_n^c]$, consider a mixing event of c individuals in a population of size n that contains nx susceptibles and ny infectives. As in the proof of Lemma 7.3, label the individuals in the mixing event $1,2,\dots,c$. For $i=1,2,\dots,c$, let $\chi_1^{(n)}=1$ if individual 1 is infected during the mixing event and $\chi_1^{(n)}=0$ otherwise, so $Z^{(n)}=\chi_1^{(n)}+\chi_2^{(n)}+\dots\chi_c^{(n)}$. By exchangeability, $\mathrm{E}[Z_n]=c\mathrm{P}(\chi_1^{(n)}=1)$. Let $S_1^{(n)}$ be the event that individual 1 is susceptible and $Y_1^{(n)}$ be the number of infectives among individuals $2,3,\dots c$. Now

$$\mathrm{P}(\chi_1^{(n)}=1|S_1^{(n)},Y_1^{(n)})=1-(1-\pi_c)^{Y_1^{(n)}}\le\pi_c Y_1^{(n)},$$

so

$$\mathrm{P}(\chi_1^{(n)}=1)\le\pi_c\mathrm{P}(S_1^{(n)})\mathrm{E}[Y_1^{(n)}|S_1^{(n)}]\le\pi_c\mathrm{E}[Y_1^{(n)}|S_1^{(n)}].$$

For $i=2,3,\dots,c$, let $\xi_i^{(n)}=1$ if individual i is infective and $\xi_i^{(n)}=0$ otherwise. Then $Y_1^{(n)}=\sum_{i=2}^c\xi_i^{(n)}$ and $\mathrm{E}[Y_1^{(n)}|S_1^{(n)}]=(c-1)\mathrm{P}(\xi_2^{(n)}=1|S_1^{(n)})$, since $\xi_2^{(n)},\xi_3^{(n)},\dots,\xi_c^{(n)}$ are exchangeable. Now $\mathrm{P}(\xi_2^{(n)}=1|S_1^{(n)})=\frac{ny}{n-1}$, so

$$\mathrm{P}(\chi_1^{(n)}=1)\le\pi_c(c-1)y\frac{n}{n-1}\le2\pi_c(c-1)y, \tag{B8}$$

whence

$$\mathrm{E}[Z_n]\le2\pi_c c(c-1)y. \tag{B9}$$

A similar argument yields $\mathrm{E}[Z|D_n^c]=c\mathrm{P}(\chi_1^{(n)}=1|D_n^c)$ and

$$\begin{aligned}\mathrm{P}(\chi_1^{(n)}=1|D_n^c)&\le\pi_c\mathrm{E}[Y_1^{(n)}|S_1^{(n)},D_n^c]\\&=\pi_c(c-1)\mathrm{P}(\xi_2^{(n)}=1|S_1^{(n)},D_n^c).\end{aligned}$$

Given that D_n^c and $S_1^{(n)}$ both occur, then either $\zeta_2^{(n)}=\zeta_1^{(n)}$, in which case individual 2 is susceptible and $\mathrm{P}(\xi_2^{(n)}=1)=0$, or $\zeta_2^{(n)}\ne\zeta_1^{(n)}$, in which case $\mathrm{P}(\xi_2^{(n)}=1)=\frac{ny}{n-1}$. Thus, $\mathrm{P}(\xi_2^{(n)}=1|S_1^{(n)},D_n^c)\le\frac{ny}{n-1}$, whence

$$\mathrm{P}(\chi_1^{(n)}=1|D_n^c)\le2\pi_c(c-1)y \tag{B10}$$

and

$$\mathrm{E}[Z|D_n^c]\le2\pi_c c(c-1)y.$$

The first inequality in (B5) now follows immediately using (B7) and the upper bound for $\mathrm{P}(D_n^c)$ given at (7.19), since $\tilde{\mu}_c^{(n)}(x,y)=y^{-1}\mathrm{E}[Z_n]$ and $\tilde{\mu}_c(x,y)=y^{-1}\mathrm{E}[Z]$.

For future reference, note that

$$\tilde{\mu}_c^{(n)}(x,y)\le2\pi_c c(c-1). \tag{B11}$$

Turning to the second inequality in (B5), by exchangeability,

$$\mathrm{E}[Z_n^2|D_n^c]=c\mathrm{P}(\chi_1^{(n)}=1)+c(c-1)\mathrm{P}(\chi_1^{(n)}=1,\chi_2^{(n)}=1)\le c^2\mathrm{P}(\chi_1^{(n)}=1)$$

and a similar argument shows that

$$\mathrm{E}[Z^2|D_n^c]\le c^2\mathrm{P}(\chi_1^{(n)}=1|D_n^c).$$

Hence, using (B8) and (B10),

$$\max(\mathrm{E}[Z_n^2|D_n^c],\mathrm{E}[Z^2|D_n^c])\le2\pi_c c^2(c-1)y.$$

The second inequality in (B5) now follows immediately using (B7), with Z_n and Z replaced by Z_n^2 and Z^2, and the aforementioned upper bound for $\mathrm{P}(D_n^c)$, since $\tilde{\mu}_{c,2}^{(n)}(x,y)=y^{-1}\mathrm{E}[Z_n^2]$ and $\tilde{\mu}_{c,2}(x,y)=y^{-1}\mathrm{E}[Z^2]$.

For future reference, note that

$$\tilde{\mu}^{(n)}_{c,2}(x,y) \leq 2\pi_c c^2(c-1). \tag{B12}$$

(b) Note that (7.27) implies that $\tilde{\mu}^{(n)}_c(x,y) = \tilde{\mu}_c(x,y)$ and $\tilde{\mu}^{(n)}_{c,2}(x,y) = \tilde{\mu}_{c,2}(x,y)$ for all $(x,y) \in \tilde{E}(y_0)$ with $y \leq 0$, so (B6) holds trivially for such (x,y). For $(x,y) \in \tilde{E}(y_0)$ with $y > 0$, (B5) and the continuity of $\tilde{\beta}_{\boldsymbol{l}}$ ($\boldsymbol{l} \in \Delta$) imply that $\tilde{\beta}^{(n)}_{\boldsymbol{l}}$ ($\boldsymbol{l} \in \Delta^{(n)}$) can be interpolated continuously between the points of E_n so that (B6) holds. □

For $n = 2,3,\dots$ and $(x,y) \in E$, let

$$\tilde{g}^{(n)}(x,y) = \sum_{c=2}^{n} p^{(n)}_C(c)\tilde{\mu}^{(n)}_c(x,y) \qquad \text{and} \qquad \tilde{h}^{(n)}(x,y) = \sum_{c=2}^{n} p^{(n)}_C(c)\tilde{\mu}^{(n)}_{c,2}(x,y).$$

Lemma B.3 *Suppose that* $C^{(n)} \xrightarrow{\mathrm{D}} C$ *as* $n \to \infty$.

(a) If $\sum_{c=2}^{n} \pi_c c^3 p^{(n)}_C(c) \to \sum_{c=2}^{\infty} \pi_c c^3 p_C(c)$ *as* $n \to \infty$ *and* $\sum_{c=2}^{\infty} \pi_c c^5 p_C(c) < \infty$*, then*

$$\lim_{n\to\infty} \sup_{(x,y)\in\tilde{E}(y_0)} \left|\tilde{h}^{(n)}(x,y) - \tilde{h}(x,y)\right| = 0. \tag{B13}$$

(b) If $\lim_{n\to\infty} \sqrt{n}\sum_{c=2}^{\infty} \pi_c c^2 |p^{(n)}_C(c) - p_C(c)| = 0$ *and* $\sum_{c=2}^{\infty} \pi_c c^4 p_C(c) < \infty$*, then*

$$\lim_{n\to\infty} \sqrt{n} \sup_{(x,y)\in\tilde{E}(y_0)} \left|\tilde{g}^{(n)}(x,y) - x\tilde{g}(y)\right| = 0. \tag{B14}$$

Proof The proof is similar to that of Lemma 7.4.

(a) Note that $p^{(n)}_C(c) = 0$ for $c > n$ and $\tilde{h}^{(n)}(x,y) = \tilde{h}(x,y)$ for all $(x,y) \notin E \setminus \{y = 0\}$. Thus

$$\begin{aligned}
\sup_{(x,y)\in\tilde{E}(y_0)} \left|\tilde{h}^{(n)}(x,y) - \tilde{h}(x,y)\right| &= \sup_{(x,y)\in E\setminus\{y=0\}} \left|\sum_{c=2}^{\infty} p^{(n)}_C(c)\tilde{\mu}^{(n)}_{c,2}(x,y) - \sum_{c=2}^{\infty} p_C(c)\tilde{\mu}_{c,2}(x,y)\right| \\
&\leq \sup_{(x,y)\in E\setminus\{y=0\}} \left|\sum_{c=2}^{\infty} \left[p^{(n)}_C(c)\tilde{\mu}^{(n)}_{c,2}(x,y) - p_C(c)\tilde{\mu}^{(n)}_{c,2}(x,y)\right]\right| \\
&\quad + \sup_{(x,y)\in E\setminus\{y=0\}} \left|\sum_{c=2}^{\infty} p_C(c) \left[\tilde{\mu}^{(n)}_{c,2}(x,y) - p_C(c)\tilde{\mu}_{c,2}(x,y)\right]\right| \\
&\leq \sup_{(x,y)\in E\setminus\{y=0\}} \sum_{c=2}^{\infty} \tilde{\mu}^{(n)}_{c,2}(x,y) \left|p^{(n)}_C(c) - p_C(c)\right| \\
&\quad + \sup_{(x,y)\in E\setminus\{y=0\}} \sum_{c=2}^{\infty} p_C(c) \left|\tilde{\mu}^{(n)}_{c,2}(x,y) - \tilde{\mu}_{c,2}(x,y)\right| \\
&\leq 2\sum_{c=2}^{\infty} \pi_c c^2(c-1) \left|p^{(n)}_C(c) - p_C(c)\right| + \sum_{c=2}^{\infty} \frac{\pi_c c^5 p_C(c)}{n},
\end{aligned} \tag{B15}$$

where at the final step we have used the bound given by (B12) in the first inequality (the functions $\tilde{\beta}_l^{(n)}$ can be defined so that inequality holds for all $(x,y) \in E \setminus \{y=0\}$) and the second inequality in Lemma B.2(b) in the second inequality. Now

$$\lim_{n\to\infty} \pi_c c^2(c-1)p_C^{(n)}(c) = \pi_c c^2(c-1)p_C(c) \qquad (c = 2,3,\dots),$$

so

$$\lim_{n\to\infty} \sum_{c=2}^{\infty} \pi_c c^2(c-1)\left|p_C^{(n)}(c) - p_C(c)\right| = 0$$

by Scheffé's lemma, since $\sum_{c=2}^{n} \pi_c c^3 p_C^{(n)}(c) \to \sum_{c=2}^{\infty} \pi_c c^3 p_C(c)$ as $n \to \infty$ by assumption and $\sum_{c=2}^{\infty} \pi_c c^3 p_C(c)$ is finite since $\sum_{c=2}^{\infty} \pi_c c^5 p_C(c) < \infty$. The latter implies that $\frac{1}{n}\sum_{c=2}^{\infty} \pi_c c^5 p_C(c) \to 0$ as $n \to \infty$, and (B13) follows.

(b) An analogous argument to the proof of part (a) yields (c.f. (B15))

$$\sqrt{n} \sup_{(x,y)\in\tilde{E}(y_0)} \left|\tilde{g}^{(n)}(x,y) - x\tilde{g}(y)\right| \leq 2\sqrt{n}\sum_{c=2}^{\infty} \pi_c c(c-1)\left|p_C^{(n)}(c) - p_C(c)\right| + \sqrt{n}\sum_{c=2}^{\infty} \frac{\pi_c c^4 p_C(c)}{n},$$

where at the final step we have used the bound given by (B11) in the first inequality and the first inequality in Lemma B.2(b) in the second inequality. The limit (B14) follows since, as $n \to \infty$, the first term on the right-hand side of (7.25) tends to 0 by assumption and the second term tends to 0 as $\sum_{c=2}^{\infty} \pi_c c^4 p_C(c)$ is finite. □

To complete the proof of Theorem 7.3 we show that the above lemmas imply that the conditions of Theorem 7.2 are satisfied. (The ODEs (3.21)–(3.23) governing the elements of the variance/covariance $\tilde{\boldsymbol{\Sigma}}(t)$ follow using (3.14).) Note that the infinitesimal drift and variance/covariance functions of $\{(\tilde{S}^{(n)}(t), \tilde{I}^{(n)}(t)) : 0 \leq t \leq t_0\}$ are given by

$$\tilde{\boldsymbol{F}}^{(n)}(x,y) = (-\lambda\tilde{g}^{(n)}(x,y), \lambda\tilde{g}^{(n)}(x,y) - \gamma) \tag{B16}$$

and

$$\tilde{\boldsymbol{G}}^{(n)}(x,y) = \begin{bmatrix} \lambda\tilde{h}^{(n)}(x,y) & -\lambda\tilde{h}^{(n)}(x,y) \\ -\lambda\tilde{h}^{(n)}(x,y) & \lambda\tilde{h}^{(n)}(x,y) + \gamma \end{bmatrix}. \tag{B17}$$

Condition (7.9) is satisfied using Lemma B.3(a), and equations (7.28) and (B17). The function $\tilde{\boldsymbol{G}}$ is bounded and uniformly continuous on $\tilde{E}(y_0)$ by Lemma B.1(b). Condition (7.10) is satisfied using Lemma B.3(b) and equations (7.28) and (B16), and $\tilde{\boldsymbol{F}}$ has uniformly continuous first partial derivatives on $\tilde{E}(y_0)$ by Lemma B.1(a)(iii). Finally, condition (7.11) is satisfied by assumption.
